# Efficient Mn^2+^ Doping in Non-Stoichiometric Cesium Lead Bromide Perovskite Quantum Dots

**DOI:** 10.1021/jacs.5c12086

**Published:** 2025-09-15

**Authors:** Lamia Hidayatova, Chenjia Mi, Novruz G. Akhmedov, Yuan Liu, Arjumand K. Shafiq, Hadi Afshari, Nishya Mohamed-Raseek, Dilruba A. Popy, Sisi Xiang, Yi-Chen Chen, Bayram Saparov, John W. Peters, Dmitri V. Talapin, Bin Chen, Madalina Furis, Evan R. Glaser, Yitong Dong

**Affiliations:** † Department of Chemistry and Biochemistry, 6187 The University of Oklahoma, Norman, Oklahoma 73019, United States; ‡ Department of Chemistry, James Franck Institute, and Pritzker School of Molecular Engineering, University of Chicago, Chicago, Illinois 60637, United States; § Homer L. Dodge Department of Physics and Astronomy, 6187 The University of Oklahoma, Norman, Oklahoma 73019, United States; ∥ Department of Materials Science and Engineering, Texas A&M University, College Station, Texas 77843, United States; ⊥ Center for Nanoscale Materials, Argonne National Laboratory, Argonne, Illinois 60439, United States; # Department of Chemistry, 3270 Northwestern University, Evanston, Illinois 60208, United States; ¶ Center for Quantum Research and Technology, The University of Oklahoma, Norman, Oklahoma 73019, United States; ○ 41487 U.S. Naval Research Laboratory, Washington D. C. 20375, United States

## Abstract

Doping magnetic transition metal ions (e.g., Mn^2+^) into colloidal quantum dots endows novel optical and magnetic properties to the host materials. CsPbBr_3_ quantum dots (QDs) are emerging light-emitting materials with high structural and chemical flexibility in the visible spectral regime. However, efficiently doping Mn^2+^ ions in CsPbBr_3_ QDs remains challenging, especially when size confinement and ensemble uniformity are needed for understanding the underexplored exciton-dopant exchange interaction. Here, we introduce a doping mechanism based on electrostatic surface Mn^2+^ adsorption that enables efficient Mn^2+^ incorporation in strongly confined CsPbBr_3_ QDs. The resultant QDs are found to have a Cs-deficient stoichiometry compared to their undoped counterparts. A redox reaction-based purification method was developed to remove Mn^2+^ cations that are tightly adsorbed on the surface to determine the concentration of lattice-incorporated Mn^2+^. Our synthesis enables a Mn^2+^ doping/alloying concentration of up to ∼44% with a Mn^2+^ photoluminescence efficiency exceeding 90%. This allows for the determination of the intrinsic exciton-to-dopant energy transfer rate.

## Graphical abstract

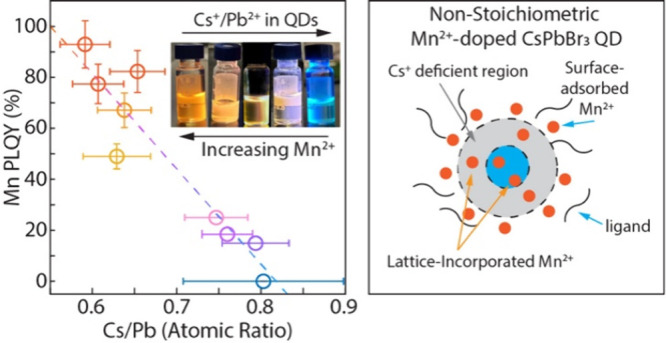

## Introduction

Incorporating impurities such as manganese­(II) in colloidal quantum dots (QDs) has been demonstrated as a versatile way to impart new optical and magnetic properties to the host material.^1–4^ Over the past decade, lead halide perovskite QDs have been explored as a new family of host materials for their highly efficient photoluminescence (PL) and facile synthesis.^5–7^ In the all-inorganic CsPbX_3_ (X = Cl, Br, I) family, Mn^2+^-doped CsPbCl_3_ QDs were first demonstrated.^8,9^ Although CsPbCl_3_ QDs often have low photoluminescence quantum yield (PLQY), benefiting from the fast exciton-to-Mn Dexter-type energy transfer, Mn^2+^-doped CsPbCl_3_ nanocrystals exhibit intense and broad emission from ^4^T_1g_ to ^6^A_1g_ d-d transitions rather than weak and sharp emission from excitons.^10–16^ The improved emission efficiency in the visible spectral range has enabled the application of CsPbCl_3_ for solar concentrators and down-converters.^17–21^

Compared to CsPbCl_3_, the band gap of CsPbBr_3_ falls into the visible spectral regime and is more suitable for solar energy harvesting and light-emitting applications. However, direct doping of Mn^2+^ ions into CsPbBr_3_ perovskite nanocrystals has been surprisingly more challenging than CsPbCl_3_ nanocrystals. Alternatively, Mn^2+^ doping in mixed-halide (Cl/Br) perovskite nanocrystals can be obtained either by introducing multiple halides in precursors during the synthesis or by converting presynthesized Mn-doped CsPbCl_3_ into CsPb­(Br/Cl)_3_ through postsynthesis Br^–^ exchange.^22^ Such approaches often result in the loss of Mn^2+^ dopants and their PL emissions when the Br^–^ composition increases. Recent studies have also revealed that Mn–Cl bonds, rather than Mn–Br bonds, are preferred in mixed-halide nanocrystals.^23^ The uncertainties in material composition and the chemical environment of Mn^2+^ add additional barriers to understanding the Mn incorporation mechanisms and exciton-Mn interaction in pure CsPbBr_3_ lattices.

Another method to introduce Mn^2+^ into the host lattices is cation exchange. While the approach has not been very fruitful for 0D CsPbBr_3_ QDs, strong Mn^2+^ PL emission can be obtained by cation-exchange in anisotropic CsPbBr_3_ nanoplatelets (NPLs), nanowires (NWs), and 2D nanoclusters.^24–30^ The large surface area and surface defects facilitate the Mn^2+^ attachment and the diffusion of Mn^2+^ into the lattices. In addition, confinement effects due to reduced dimensionality increase the bandgap, promoting exciton-to-dopant energy transfer for enhanced Mn emission. However, such postsynthesis treatments often yield significant variations in particle lateral sizes, which can be intensified by Ostwald ripening during the treatment. Additionally, the Mn^2+^ dopant can still migrate or be expelled from the nanoparticles. It is worth noting that the one-pot synthesis of Mn-doped low-dimensional nanostructures at room temperature has also been demonstrated.^29,31–33^ Despite the successes of Mn^2+^ doping in these anisotropic nanostructures, the lack of ensemble uniformity and uncertainties on dopant distribution in each particle have hindered the understanding of exciton-dopant exchange interaction from the ensemble-averaged optical properties.

Direct hot-injection synthesis of 0D Mn-doped CsPbBr_3_ QDs usually produces weakly quantum-confined or irregularly shaped nanoparticles.^34^ The relatively small host bandgap leads to a low Mn^2+^ emission efficiency.^8^ The bandgap of CsPbBr_3_ QDs can be expanded to ∼ 2.7 eV by reducing the QD size.^35–37^ Furthermore, strong 3D confinement forces the spatial overlap between the wave functions of the Mn dopants and the exciton, promoting Mn^2+^ PL efficiencies.^8,38^ Unfortunately, there have been limited attempts to directly dope Mn^2+^ in strongly confined CsPbBr_3_ QDs adopting hot-injection synthesis for high ensemble uniformity. A pioneering study suggests that the high Mn–Br bond dissociation energy makes the decomposition of Mn–Br precursors difficult, subsequently reducing doping efficiency.^8^ In addition, incorporating Mn^2+^, a hard Lewis acid, into QD lattices composed of soft Lewis acid and base (Pb^2+^ and Br^–^) will be less preferred than doping Mn^2+^ in CsPbCl_3_ with a hard Lewis base (Cl^–^).^23^ The thermodynamics of Mn-X bonds have certainly affected the ability to synthesize Mn^2+^-doped CsPbBr_3_ QDs. To date, improved Mn^2+^ PL emission in directly hot-injection synthesized Mn^2+^-doped CsPbBr_3_ QDs (∼6.5 nm) has been demonstrated as a side product through the conversion of intermediate Mn^2+^-doped 2D L_2_PbBr_4_ plates into Mn^2+^-doped QDs and NPLs.^39^ Most recently, room temperature Mn^2+^ doping in 5 nm CsPbBr_3_ QDs has also been demonstrated.^31^ Nevertheless, the size regulation effect and Mn^2+^ incorporation efficiency or dopant density are still limited. Therefore, a generalized doping method is needed to produce monodispersed QDs with high doping efficiency in order to understand the photophysics of exciton-Mn interaction in CsPbBr_3_ QDs.

In this work, we developed a synthesis under a bromide-rich environment with high Mn^2+^ ionic strength for efficient Mn^2+^ doping in size-confined CsPbBr_3_ QDs. The resulting Mn^2+^-doped CsPbBr_3_ QDs (∼4 nm) exhibit efficient (90 ± 10% PLQY) Mn^2+^ emission with a doping/alloying concentration up to ∼44%. These Mn^2+^-doped QDs are highly nonstoichiometric, featuring Cs-deficient regions near the QD surface. The Mn^2+^ doping efficiency increases with the extent of the Cs deficiency. Nuclear magnetic resonance (NMR) spectroscopy and elemental analysis suggest that a large quantity of Mn^2+^ ions is tightly adsorbed on the QD surface, which can be thoroughly removed by chemical redox-reaction-based purification using H_2_O_2_ and HBr without affecting the Mn^2+^ ions incorporated in the QD lattices. Finally, the exciton-to-dopant energy transfer rate is measured by transient absorption (TA) spectroscopy. Our study provides a facile way to dope Mn^2+^ in perovskite QDs, providing new insights to apply perovskite QDs to light-harvesting, spintronics, and hot electron production applications.^40^

## Results

Mn^2+^-doped CsPbBr_3_ QDs were prepared using manganese­(II) acetate tetrahydrate as the Mn^2+^ precursor in a one-pot synthesis (details in Experimental Section). The bromide-rich environment is created by adding hydrobromic acid (HBr aqueous solution) to the system, leading to a bromide-terminated QD surface.^35,41^ HBr also reacts with Mn acetate tetrahydrate (Mn­(Ac)_2_·4H_2_O) to facilitate the decomposition of Mn^2+^ precursors by producing acetic acid that can be removed from the system after extended evacuation. The Mn^2+^ incorporation efficiency can be tuned by solely varying the loading amount of Mn­(Ac)_2_·4H_2_O/HBr (more details of the synthesis conditions and their effects on Mn^2+^ doping are provided in the Discussion section, Supporting Information Table S1, Supporting Information Note 1, and Figure S1).

Absorption and PL spectra of the Mn^2+^-doped and undoped (control) QDs at room temperature are plotted in Figure 1a and 1b. The overall PLQY of Mn^2+^-doped CsPbBr_3_ QDs (PL emissions from both exciton and Mn) is generally above 90% (Figure S2a), and the size of both Mn^2+^-doped and undoped QDs is ∼4 nm (Figure 1c and 1d, Figure S3) with a tight size distribution (±7.4% and ± 6%, respectively). Compared to undoped QDs, Mn^2+^-doped QDs tend to be more irregularly shaped. The Mn^2+^-doped QDs show an almost completely quenched exciton PL. By integrating the PL peaks from Mn^2+^ (∼605 nm), the highest Mn^2+^ emission PLQY is determined to be >90%. The exciton PL exhibits a blueshift of ∼50 meV in doped QDs compared to undoped QDs sharing similar sizes (Figures S4 and S5). This phenomenon has been observed in II–VI QDs and lead halide perovskite NCs, which can be attributed to the possible increase in bandgap resulting from Mn^2+^ alloying and local lattice periodicity breaking.^42–44^ Additionally, Mn^2+^incorporation significantly blueshifts (∼186 meV) the absorption spectrum of the QDs (see Figure 1a) and changes the profile of higher-order exciton absorptions (black vertical arrows), suggesting that Mn^2+^ alloying may have resulted from efficient dopant incorporation. No anisotropic nanostructures, such as NPLs and NWs, are produced during the synthesis, implying a doping mechanism different from the previously reported monolayer perovskite-mediated doping.^39^

**Figure 1:**
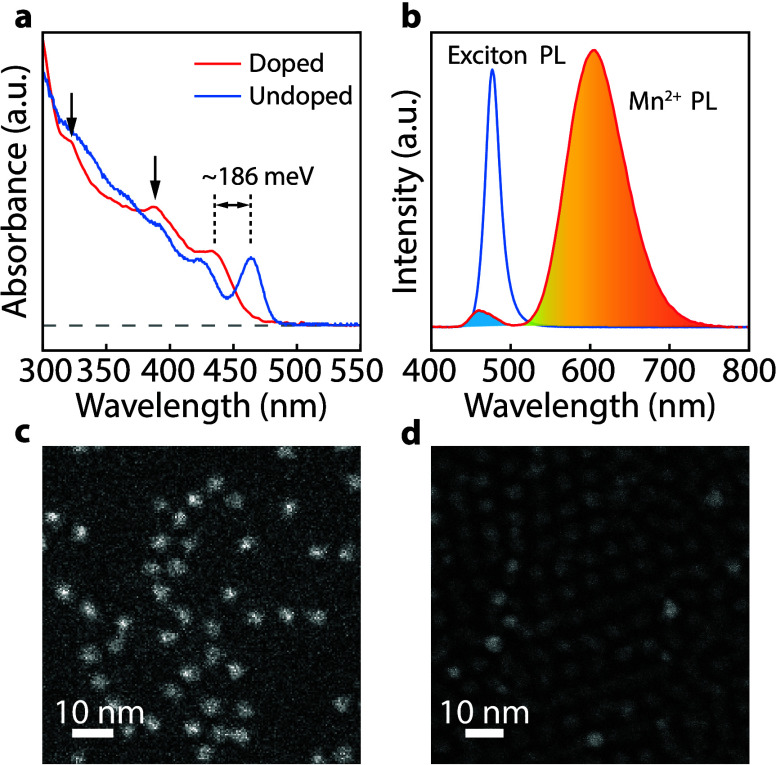
(a) Absorption spectra of Mn^2+^-doped and undoped CsPbBr_3_ QDs measured at room temperature. Vertical arrows denote higher-order exciton peaks. (b) PL spectra of Mn^2+^-doped and undoped CsPbBr_3_ QDs (Room temperature, excited using a 385 nm LED). (c) High-angle Annular Dark-field Scanning Transmission Electron Microscopy (HAADF-STEM) images of Mn^2+^-doped CsPbBr_3_ QDs (purified by the GPC-chemical-GPC approach, as discussed in the main text and details given in the Experimental Section), and (d) undoped CsPbBr_3_ QDs.

Inductively coupled plasma mass spectrometry (ICP-MS) elemental analysis is typically used to determine the chemical composition and doping concentration of QDs. Accurate determination of chemical composition in perovskite QDs is usually challenging due to the inefficient removal of unreacted precursors.^25^ Following previous reports, we purified the Mn^2+^-doped QD colloids using gel permeation chromatography (GPC) columns^25,45^ after traditional antisolvent precipitation/resuspension cycles (Figure S6). The cationic composition (atomic ratios, normalized to Pb^2+^) of QDs is then obtained from ICP-MS (Supporting Information Table S2). The Br/Pb ratio of QD was determined using energy-dispersive X-ray spectroscopy (EDS) (Figure S7 and S8) and X-ray photoelectron spectroscopy (XPS). The chemical composition (not including Mn^2+^) is Cs_0.58–0.66_PbMn_*x*_Br_3.5–4.8_ for doped QD and Cs_0.73–0.9_PbBr_3.2–4_ for undoped QDs. The bromide-rich QD composition in both QDs is expected since an anion-rich synthesis environment is used.

The Cs/Pb ratio of Mn^2+^-doped QDs with various Mn^2+^ PLQYs and undoped QDs is plotted in Figure 2a. The Cs/Pb stoichiometry of undoped QDs (0.7–0.9) matches that of the reported model structure with the QD’s surface terminated by [PbBr_2_/ABr] (A = cationic ligand) (Supporting Information Note 2).^46^ In this model, surface undercoordinated Cs^+^ ions were replaced by cationic ligands, such as oleylammonium, resulting in a Cs/Pb atomic ratio of less than one as the QD size decreases. The oleylammonium bromide surface passivation is supported by the NMR spectrum (Figure S9). In contrast, the Cs/Pb ratio in Mn^2+^-doped QDs is consistently reduced to approximately 0.6 (as low as 0.55) with increased Mn^2+^ doping, indicating a Cs deficiency in the QD lattices. Note that the sizes of all Mn^2+^-doped QDs with high Mn PLQYs (>40%) are similar (4–4.5 nm). Therefore, the size effect on the Cs/Pb ratio in these QDs is negligible. For doped QDs with lower Mn PLQY (<30%), the size can be slightly larger, which may contribute to a slightly higher Cs/Pb ratio. It is also worth noting that a considerable number of Pb^2+^ ions are replaced by Mn^2+^ in heavily doped QDs. Therefore, the actual Cs^+^ loss can be underestimated in heavily doped QDs by calculating the Cs/Pb atomic ratios.

**Figure 2:**
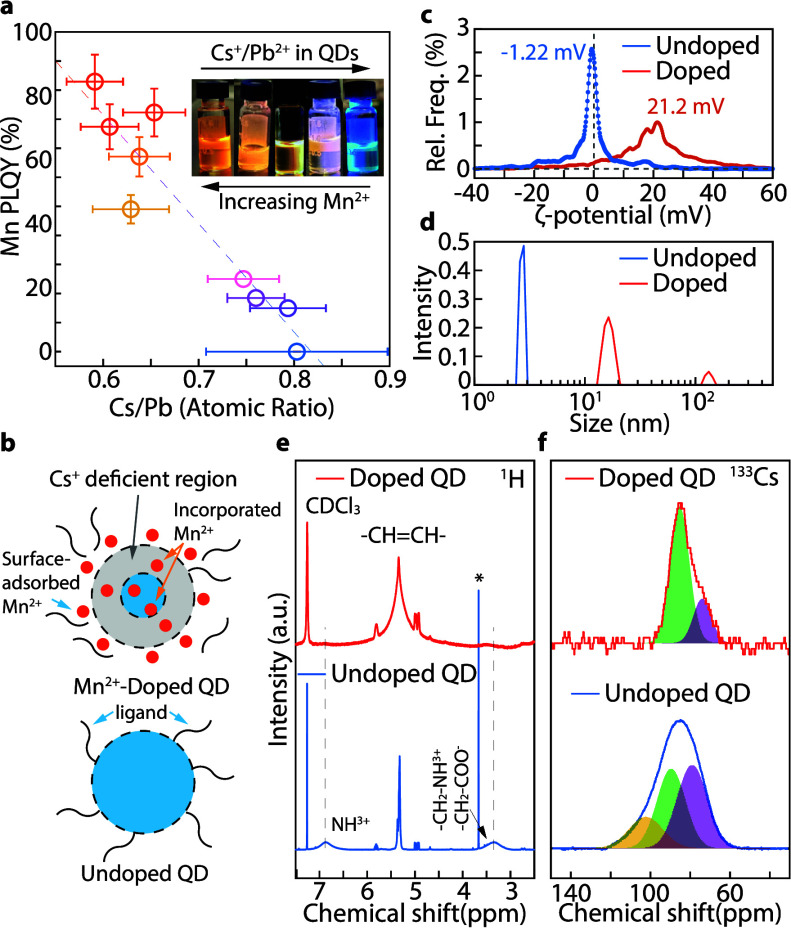
(a) Mn^2+^ PLQY of Mn^2+^-doped and undoped CsPbBr_3_ QDs with respect to the Cs/Pb ratio. Decreasing the Cs/Pb atomic ratio enhances the nonstoichiometry and Mn^2+^ emission (All QDs have overall PLQYs > 70%). Additional photographs are provided in Figure S11. (b) Schematic representation of the distribution of Mn^2+^ ions and ligands in relation to the amount of Cs. (c) Zeta-potential (ζ-potential) and (d) Dynamic light scattering (DLS) intensity-based hydrodynamic size distribution (nm) for Mn^2+^-doped and undoped CsPbBr_3_ QD colloids (purified by antisolvent only). DLS correlation functions are provided in Supporting Information Figure S12. (e) Expanded region of ^1^H NMR spectrum of Mn^2+^-doped and undoped CsPbBr_3_ QDs in chloroform-d (CDCl_3_). (f) ^133^Cs NMR signals for Mn^2+^-doped CsPbBr_3_ QDs and their fits showing core (green) and intermediate (purple) Cs^+^ species, alongside undoped CsPbBr_3_ QDs display core (green), intermediate (purple), and surface (yellow) Cs^+^ species.

Elemental analyses also showed that the Mn/Pb atomic ratio is 10–50 in almost all Mn^2+^-doped QD samples, even after GPC purification. Given the sophisticated purification processes, such an unexpectedly high Mn^2+^ concentration in QD colloids cannot be simply attributed to free-standing Mn^2+^ precursors. Instead, it suggests that many Mn^2+^ ions are tightly attached to QD surfaces, and some of them are probably bound to the surface as Z-type ligands. The extremely large excess Mn^2+^ concentration (up to ∼17,000 Mn^2+^ per QD) is beyond the capacity of typical surface ligand coverage, suggesting the majority of the excess Mn^2+^ ions are physically adsorbed on the surface of the QD. EDS and XPS measurements also confirm the large Mn/Pb ratio in the doped QDs (Figures S8 and S10). Additionally, the XPS results indicate that the doped QD sample contains a mixture of long-chain hydrocarbon moieties (oleylammoniums and oleates), with their quantity larger than that of the undoped QDs. This suggests that many excess Mn^2+^ ions exist as Mn carboxylates, such as Mn-oleates. As illustrated in Figure 2b, an electric double layer structure is proposed, in which QDs are charged due to the nonstoichiometry, and Mn^2+^ ions can electrostatically adsorb on the charged QD surface, forming a diffusion layer that balances the charge on the QDs and stabilizes the QD colloids.

The electric double layer structure is evidenced by a ζ-potential of ∼21 mV in Mn^2+^-doped QD colloids (Figure 2c). The positive ζ-potential provides further evidence that many surface-physiosorbed Mn^2+^ ions are firmly attached to QDs and cannot be easily removed by conventional purification methods. In comparison, the undoped QDs have a negligible ζ-potential value (Figure 2c). To better study the colloidal structure of our Mn^2+^-doped QDs, we measured their hydrodynamic diameters (*D*_h_) using dynamic light scattering (DLS). For undoped QDs, *D*_h_ is slightly smaller but close to the physical size of QD-ligands (∼4 nm, Figure 2d). The difference between *D*_h_ and the size measured using STEM imaging is attributed to the uncertainties in refractive indices when estimating the size distribution using the correlation function shown in Figure S12. In stark contrast, the *D*_h_ of Mn^2+^-doped QDs is ∼15 nm with a small population at ∼130 nm. The increased *D*_h_ is attributed to electric double layers and QD aggregations induced by the QD surface charges and lack of direct organic ligand passivation in Mn^2+^-doped QDs. STEM images also confirm that the Mn^2+^-doped QDs can aggregate (Figure S13). The surface Mn^2+^ adsorption on QDs plays an important role in facilitating Mn^2+^ incorporation in CsPbBr_3_ lattices, given that perovskite QD surface ions are often under dynamic solubility equilibria. Details of the proposed mechanism for efficient Mn^2+^ doping are described in the Discussion section.

^1^H NMR spectroscopy was used to study the chemistry of Mn^2+^-doped QD surfaces. Figure 2e (blue) shows that undoped QDs are capped by alkylammonium cations, which exhibit broadened peaks (indicated by the dashed vertical lines) at 3.34 ppm (alpha protons) and 6.87 ppm (ammonium protons) from oleylammoniums, in good agreement with previous studies.^47–49^ However, the characteristic peak of alkylammonium alpha-protons nearly disappears in the ^1^H NMR spectrum of Mn^2+^-doped QDs. Additionally, the peak from the alkenyl protons at 5.4 ppm in oleate anions and oleylammonium cations is significantly broadened (Figure 2e (red)). The line broadening is attributed to the effect of the paramagnetic Mn^2+^ ions adsorbed on the QD’s surface, forming ion pairs with organic oleates. The oleates are counterions for surface-adsorbed Mn^2+^, imparting the colloidal stability of doped QDs in organic solvents such as toluene and hexanes. The interaction of the paramagnetic Mn^2+^ ions with surrounding molecules shortens the spin–spin relaxation time (T_2_), which in turn leads to the broadening of the peaks (given that the line width is inversely proportional to T_2_ ( $\upsilon_{1/2}=\frac{1}{{\pi T}_{2}}$ ). The NMR results agree well with our QD surface structural model.

The nonstoichiometry of Mn^2+^-doped QDs is further supported by the ^133^Cs NMR spectrum (Figure 2f, top). In the undoped QD (Figure 2f, bottom), the ^133^Cs signal is broadened because Cs^+^ ions are distributed in different regions in the QDs with various lattice disordering. The spectrum can be fitted with a minimum of three Gaussian peaks, corresponding to three groups of Cs^+^ ions depending on their locations in the QDs and on the degree of lattice disorder: the lattices in the core (least disordered), the lattices on or very close to the surface (most disordered), and the lattices in the intermediate region. This is in good agreement with a previous study using well-passivated CsPbBr_3_ QDs.^50^ The ^133^Cs signal of the Mn^2+^-doped QDs is much narrower (Figure 2f, top) due to, in particular, the absence of the downfield (yellow-shaded) component associated with the Cs^+^ ions located in the surface lattices. This suggests that the nonstoichiometric Mn^2+^-doped QD has Cs deficient lattices on and near the surface, promoting surface Mn^2+^ adsorption. The ^1^H and ^133^Cs NMR spectra of Mn^2+^-doped QDs with lower Mn^2+^ PLQY show less peak narrowing and a surface Cs component smaller than undoped QDs (Figure S14). The line width changes observed in the ^133^Cs NMR spectrum corroborate the correlation between the extent of Cs deficiency and Mn^2+^ doping efficiency.

Excess Mn^2+^ ions in the QD colloid must be removed to accurately quantify the Mn^2+^ incorporation. Since the surface-adsorbed Mn^2+^ is not incorporated into the crystal lattices, we employed a chemical purification method by oxidizing Mn^2+^ using H_2_O_2_ and removing the generated MnO_*x*_ using hydrobromic acid (Details in the Experimental Section). Figure 3a shows the absorption and PL spectra of a Mn^2+^-doped QD sample before chemical purification, after H_2_O_2_ oxidation, and after HBr treatment. No noticeable spectral shifts or Mn^2+^ PL intensity changes are found, indicating that the surface-adsorbed Mn^2+^ ions are not emissive, and the lattice incorporated Mn^2+^ ions are not oxidized. However, after H_2_O_2_ treatment, the QD solution becomes darker in color (Figure 3b) due to the generation of MnO_*x*_, which tends to be detached from the QD surface. HBr aqueous solution was then added to the QD solution to reduce MnO_*x*_ and extract the produced Mn^2+^ into the aqueous phase. After cleaning, the QD solution returns to its light-yellow color. It is worth noting that extensive iterations of H_2_O_2_/HBr purification will decrease the Mn^2+^ emission intensity and compromise the QD colloidal stability (Figure S15). After chemical purification, the QDs were purified again using GPC to remove any possible free-standing Mn^2+^.

**Figure 3:**
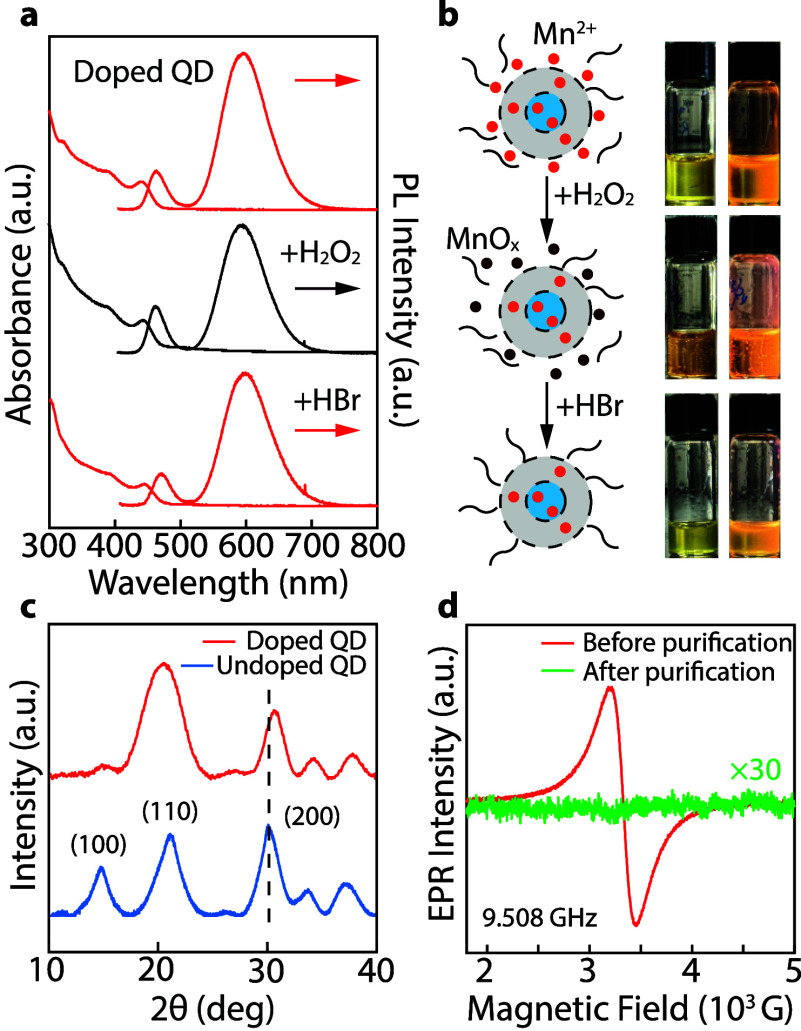
(a) Absorption and PL spectra of Mn^2+^-doped QD and surface purification with H_2_O_2_ and HBr. The intensity of the PL spectra in (a) is normalized with respect to the samples’ absorbance values at 385 nm. (b) Schematic diagrams illustrating Mn^2+^-doped QDs before, during the oxidation of Mn^2+^, and after the removal of surface-adsorbed Mn^2+^ ions, accompanied by photographs taken in daylight (left column) and under UV light (right column). (c) XRD patterns of the Mn^2+^-doped (red curve) and undoped (blue curve) CsPbBr_3_ QDs. A noticeable shift of the (200) XRD peak to higher 2θ values results from lattice contraction as Mn^2+^ ion concentration increases, expected from substituting larger Pb^2+^ ions with smaller Mn^2+^ ions. (d) EPR spectra of Mn^2+^-doped CsPbBr_3_ QDs before and after GPC-chemical-GPC purifications at room temperature.

Electron paramagnetic resonance (EPR) spectroscopy was also employed to investigate the different chemical environments of Mn^2+^ ions in the Mn^2+^-doped QD colloids (Figure 3d). The EPR spectrum of Mn^2+^-doped QD colloids after only antisolvent purifications exhibits strong EPR signal with a full width at half-maximum (fwhm) of ∼235 G (Figure 3d, red curve). No hyperfine structure was observed. Based on the elemental analyses above, the EPR response of Mn^2+^-doped QD samples before purification is likely dominated by the surface-adsorbed Mn^2+^. In fact, a very similar EPR spectrum is obtained from a Mn-oleate solution (Figure S16), also aligning with a previous report.^23^ Interestingly, after GPC-chemical-GPC purification, the EPR signal of the Mn^2+^-doped QD colloids almost vanished (Figure 3d, green curve). The absence or weakness of EPR responses from lattice-incorporated Mn^2+^ in Mn^2+^-doped CsPbBr_3_ NCs was attributed to strong antiferromagnetic coupling in linear Mn–Br–Mn bonds.^23^ Given the high lattice incorporated Mn^2+^ density in our Mn^2+^-doped QDs, it is reasonable that the average distance between Mn^2+^ ions is very small, leading to very broad or nearly nondetectable EPR signals. In lightly doped QDs where dopants are more separated, weak and broad EPR responses with a fwhm of ∼600 G were found after decoupling the sharp EPR signals of surface-adsorbed Mn^2+^ from the overall EPR responses (this sample is not chemically purified due to the low concentration of surface-adsorbed Mn^2+^, which makes it unstable in harsh chemical environments, Figure S17). In addition, we note that similar broad EPR signals were reported for 0.5% and 3% Mn-doped CsPbI_3_ QD samples, which were also attributed to substantial antiferromagnetic coupling of the Mn^2+^ ions.^51^

To test the effectiveness of physiosorbed Mn^2+^ removal through the GPC-chemical-GPC purification, QD samples synthesized using the highest Mn-precursor loading ratio were used for low-temperature EPR analysis (Figure S18). After the GPC-chemical-GPC purification, QDs exhibit continuously undetectable EPR responses even at low temperatures (120 K). In contrast, similar QDs, only after antisolvent precipitation-resuspension cycles, show strong EPR signals at both room and low temperatures (Figure S18). It has also been reported that reasonably solvated Mn^2+^ species should show readily detectable EPR signals at low temperatures.^23^ The EPR results strongly suggest the thorough removal of surface-adsorbed Mn^2+^ species. This is also corroborated by ICP-MS analysis of GPC-chemical-GPC purified QDs, which shows a significantly decreased Mn/Pb atomic ratio of less than 1 compared to 40–45 before the GPC-chemical-GPC purification. The Cs/Pb ratio remained almost unchanged (0.58), indicating that the structure of the QDs remains nearly intact after purification. Furthermore, we have quantified the spin concentration in the QD samples before GPC-chemical-GPC purification (Figure S19). The resultant 2.9 × 10^20^ spins/mL corresponded to a Mn^2+^ concentration that matches very well with the concentration of removed Mn^2+^ in this sample determined by ICP-MS (details in Supporting Information Note 3). These results corroborated the conclusion that almost all spins in the EPR spectrum came from surface physiosorbed Mn^2+^, which were nearly all removed by the GPC-chemical-GPC purification.

Knowing that the surface physiosorbed Mn^2+^ can be fully removed, a correlation between Mn^2+^ PLQY and doping concentration in Mn^2+^-doped QDs was established by performing ICP-MS analyses on a series of Mn^2+^-doped QD samples purified by the GPC-chemical-GPC cycles (Figure S20). The doping/alloying concentrations of QDs, assuming only Pb^2+^ ions were replaced by Mn^2+^, range from ∼6.5% to ∼44%. The Mn^2+^ PLQY increases sublinearly with the doping concentration, implying Mn^2+^ dopants are not optically equivalent when the doping/alloying concentration is high. The efficient incorporation of Mn^2+^ in CsPbBr_3_ QDs was also confirmed using X-ray diffraction (XRD) measurements. As shown in Figure 3c, the (200) reflection from the XRD pattern of Mn^2+^-doped QDs experienced a 0.60° shift to higher angles due to the lattice constraint induced by replacing Pb^2+^ with smaller Mn^2+^. According to Vegard’s law (Supporting Information Note 4),^52^ the Mn^2+^ doping concentration is estimated to be 19%, falling into the range of the estimated doping concentration from the ICP-MS analysis.

Surface-adsorbed Mn^2+^ plays an important role in the luminescence stability of QDs. Studies show that lattice-incorporated Mn^2+^ tends to diffuse to the surface and leave the crystal.^53^ The PL spectra of an Mn^2+^-doped QD colloid (Figure 4a) show virtually no intensity drops after five iterations of precipitation-resuspension purification cycles using methyl acetate as the antisolvent. In comparison, an undoped QD colloid experiences a PLQY efficiency drop from ∼80% to <20% after two iterations of purification (Figure S21a). Additionally, the Mn^2+^-doped QD showed no noticeable PLQY decrease over 120 days of storage (Figure S21b). The outstanding Mn^2+^ PL stability can be attributed to the protection of surface Mn^2+^ adsorption, which suppresses the diffusion loss of doped Mn^2+^. Indeed, after GPC-chemical-GPC purification, Mn^2+^-doped QDs exhibit reduced but adequate Mn^2+^ PL stability (Figure S22a and S22b). To better reveal the function of surface-adsorbed Mn^2+^ and the stability of lattice-incorporated Mn^2+^ ions, we have studied the thermal stability of Mn^2+^ PL emission using QDs before and after GPC-chemical-GPC purifications. Specifically, the PL spectra of QD colloids were monitored over time at 80 °C (Figure S22c and S22d). A QD solution with surface-adsorbed Mn^2+^ exhibits an ∼20% Mn^2+^ PL intensity drop at 600 nm, whereas purified QD experienced a ∼50% Mn^2+^ PL intensity drop. While the PL drop can be partially attributed to heat-induced nonradiative processes that compete with exciton-to-dopant ET, the additional PL loss in the purified sample can be explained by the loss of Mn^2+^, presumably located in the surface lattices (or close to) of the QDs. Interestingly, the Mn^2+^ PL intensity drop of purified QDs became very slow after 5 min, indicating that some Mn^2+^ ions incorporated in QD lattices are more stable than others. The two categories of Mn^2+^ dopants coincide with the two different types of lattices in QDs: surface lattices with Cs deficiency and center/intermediate lattices with normal stoichiometry. Further thoughts on the potential correlations between Mn^2+^ doping locations and their chemical environments are given in the Discussion section.

**Figure 4:**
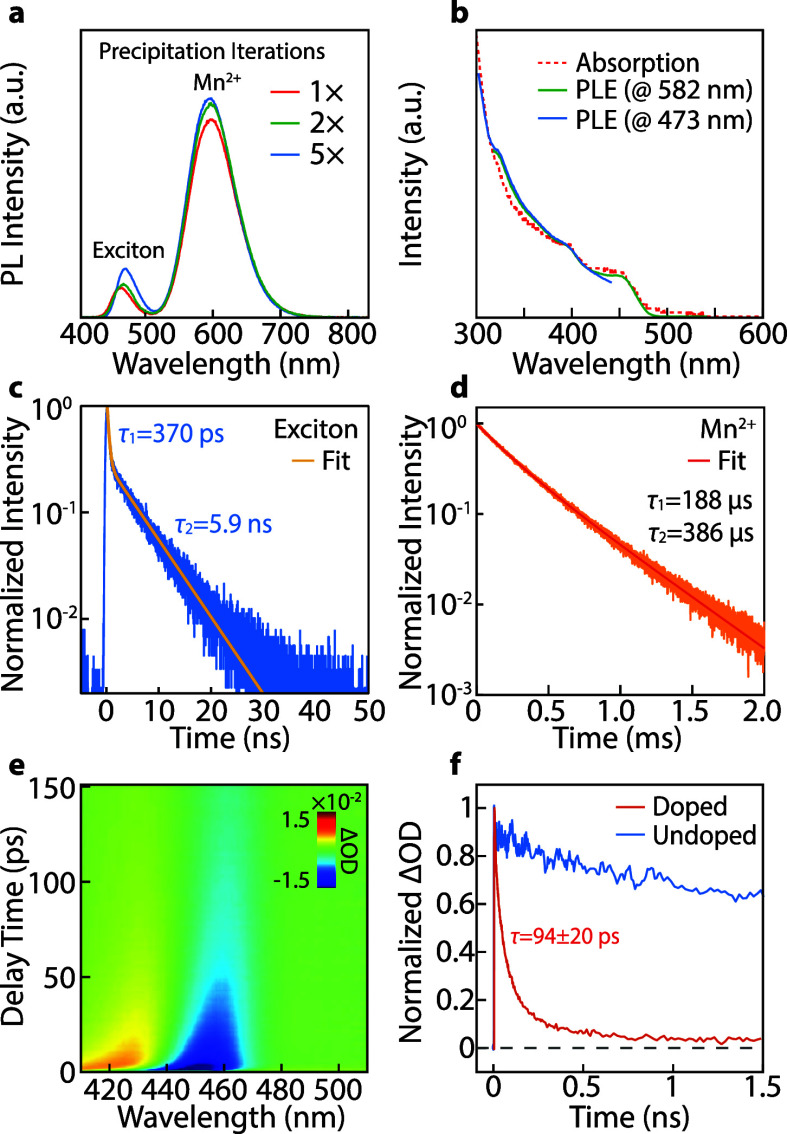
(a) PL spectra of Mn^2+^-doped QDs after 1×, 2×, and 5× antisolvent precipitation-dissolution iterations using methyl acetate as the antisolvent. (b) Absorption (dotted red curve) and PLE spectra of Mn^2+^-doped QD were monitored at 582 nm (green curve) and 473 nm (blue curve), respectively. (c) The decay curve of exciton emission for Mn^2+^-doped CsPbBr_3_ QDs. (d) Phosphorescence decay curves recorded at 600 nm show the Mn^2+^ emission from Mn^2+^-doped CsPbBr_3_ QDs (Mn^2+^ PLQY ∼75%). The solid lines in (c) and (d) are fits using the biexponential decay functions. (e) 2D contour plot of TA spectra of Mn^2+^-doped CsPbBr_3_ QDs with pump intensities of ∼18.8 μJ/cm^2^. (f) Bleach recovery dynamics in TA spectra of undoped (blue, monitored at 470 nm) and Mn^2+^-doped (orange, monitored at 450 nm) CsPbBr_3_ QDs.

Photoluminescence excitation (PLE) spectra obtained at two different wavelengths (473 and 582 nm) from the exciton and Mn^2+^ PL overlap with the absorption spectrum of Mn^2+^-doped QDs, suggesting all Mn^2+^ emission bands are sourced from excitons in QDs (Figure 4b). To understand the recombination dynamics of excitons and Mn^2+^ dopants in CsPbBr_3_ QDs, we performed time-resolved PL intensity measurements. The PL decay trace of the exciton emission can be fitted by a biexponential decay function with a ∼370 ps fast component (convoluted with the instrument response function) and a 5.9 ns slow component (Figure 4c). Given the high PLQY of Mn^2+^-doped QDs, we attribute the fast decay to energy transfer (ET) or possible electron transfer, and the slower decay to exciton radiative recombination from a small fraction of undoped QDs or QDs with insufficient dopant incorporation in the ensemble. The temporal evolution of the Mn^2+^ emission intensity is plotted in Figure 4d. Considering the two categories of Mn^2+^ in lattices near the surface and in the core, the Mn^2+^ PL decay curve was fit by a biexponential decay function with time constants of 188 and 386 μs (the fitting parameters and a single exponential fit are provided in Figure S23 and Table S3). Such a biexponential PL decay is more evident in QDs with lower doping levels (Figure S24). The two decay components are tentatively attributed to Mn^2+^ dopants in two regions with different degrees of interdopant interaction. In comparison, the Mn^2+^ emission lifetime can be as long as ∼2 ms and 300–400 μs when the dopants are diluted in methylammonium (MA) lead bromide single crystals and CsPbBr_3_ NPLs, respectively.^28,54,55^ Given that the heavy atom effect should be similar in these two crystals, the shortened lifetime can be attributed to the Mn–Mn couplings. Indeed, reducing the Mn^2+^ doping level leads to an overall slower Mn^2+^ PL decay (Figure S24). However, two decay components can still be observed, indicating that the two possible categories of Mn^2+^ local environments are likely inherited from the doping mechanism.

Although reported for Mn^2+^-doped CsPbCl_3_ NCs,^56^ intrinsic ET rates remain elusive in 0D Mn^2+^-doped CsPbBr_3_ QDs. TA spectroscopy was used to determine the ET rate in Mn^2+^-doped QDs. Figure 4e and 4f show the TA spectra and the bleach recovery dynamic traces monitored at the peak position for undoped and Mn^2+^-doped CsPbBr_3_ QDs, respectively. Both samples exhibit a PLQY greater than 75%, indicating that the exciton trapping process has a minimal impact on the bleach recovery dynamics. The fast bleach recovery in Mn^2+^-doped QDs is therefore attributed to the exciton-to-dopant ET. It is worth noting that possible exciton-to-dopant charge transfer (CT) is not ruled out in this study. CT from excitons to Mn^2+^ has been discovered in Mn-doped lead halide perovskites.^32,57,58^ We will, however, focus our discussions on ET since a recent study has revealed that CT is more preferred in lead chloride perovskites.^57^ The apparent ET rate is then extracted using reported methods to obtain a time constant of ∼94 ps.^56^ Unlike large NCs and 2D NPLs with uncertain degrees of exciton Mn^2+^ wave function overlapping, all lattice-incorporated Mn^2+^ are presumably able to couple with the exciton in these strongly confined QDs. From the estimated doping concentration of the sample (103–188 Mn^2+^ ions per QD, considering uncertainties in PLQY measurements, see Figure S20), the intrinsic exciton-to-Mn ET rate in Mn^2+^-doped CsPbBr_3_ is calculated to be 0.06–0.1 ns^–1^ per Mn^2+^. This rate, even enhanced by strong quantum confinement, is 30–50 times slower than that found in Mn^2+^-doped CsPbCl_3_ and >100 times slower than that in Mn^2+^-doped CdS and CdSe QDs.^38,56,59^

The relatively slow ET rate in Mn^2+^-doped CsPbBr_3_ QDs is unlikely to be caused by Mn-to-exciton back ET, given the large difference between the exciton and Mn^2+^ PL energies (∼630 meV). Although the ET rate can be influenced by the high ionicity of Mn^2+^ in perovskite lattices and the lower exciton energy, the significantly slower ET rate in Mn^2+^-doped CsPbBr_3_ QDs compared to that in Mn^2+^-doped CsPbCl_3_ QDs still suggests that the Mn-exciton exchange interaction is relatively weak in Mn^2+^-doped CsPbBr_3_ QDs. A previous study also indicates that the Mn-exciton exchange coupling in Mn^2+^-doped CsPbI_3_ QDs is unexpectedly weak.^51^ Several factors can contribute to the slow average ET rate in CsPbBr_3_ QDs. First, the potential Mn^2+^ dopant clustering can also introduce MnBr_4_^2–^-rich domains inside the QD, promoting exciton localizations and decreasing exciton-Mn wave function overlapping.^44^ Second, the recently reported^43,44^ lattice periodicity-breaking effect in Mn^2+^-doped CsPbBr_3_ QDs can also localize excitons to nondopant sites. Last but not least, Mn^2+^ dopants experience different lattice stoichiometries, and they may not share the same exciton-dopant interaction. To provide some insights on this, the ET rate is also determined for Mn^2+^-doped CsPbBr_3_ QDs exhibiting ∼53% and ∼7% Mn^2+^ PLQYs (Figure S25). Interestingly, a faster per Mn^2+^ ET rate is found in the lightly doped sample. This observation is qualitatively consistent with the observation that the Mn^2+^ PLQY increases sublinearly with doping concentration (Figure S20), which suggests that Mn^2+^ in different lattice environments may not exhibit the same ET kinetics. (More details are provided in Supporting Information Note 5.)

## Discussion

The doping of QDs is inherently hindered by the “self-purification” mechanism, whereby impurities are repelled toward the surface during nanocrystal growth.^60^ Self-purification makes smaller QDs even harder to dope, given that the impurity formation energy increases with increasing quantum confinement.^61^ In the case of perovskites, highly ionic lattices lead to large ion migration mobilities.^23,53^ This will accelerate the exclusion of Mn^2+^ dopant in perovskites. It has been well-established that the doping efficiency of QDs highly depends on the surface adsorption of dopant ions.^60^ To incorporate cationic substitutional impurities, an anion-rich host will facilitate surface adsorption of cationic impurities for enhanced doping efficiency. While demonstrated in conventional II–VI QDs,^61^ such a surface-adsorption facilitated doping process has not been well understood in perovskite QDs, presumably due to their dynamic surfaces that are unlikely to stabilize surface-adsorbed ions.

We propose a doping mechanism considering the surface adsorption of Mn^2+^ and stoichiometry deviations in our Mn^2+^-doped QDs. As illustrated in Figure 5, the bromide-rich surface will attract cations near the surface at the early stage of QD growth. Previous studies show that Pb^2+^ tends to form anionic complexes such as PbBr_3_^–^ and PbBr_4_^2–^.^36,41^ Therefore, Mn^2+^ and Cs^+^ are likely the main cationic species in the reaction mixture. When the divalent Mn^2+^ ions are readily available in large quantities (Mn^2+^/Cs^+^ ∼3.6, molar ratio), their surface adsorption is electronically favored, and the Mn^2+^ incorporation becomes efficient. In contrast, monovalent Cs^+^ surface adsorption is not favored. Therefore, further growth of the QD will result in Cs-deficient lattices close to the surface of QDs, as evidenced by ^133^Cs NMR spectroscopy. This Cs deficiency will subsequently encourage the surface physisorption of Mn^2+^ for charge neutrality. This can also explain why our QDs have firmly adsorbed Mn^2+^ ions that are >10-fold equivalent to the Pb^2+^ ions that constitute the QDs.

**Figure 5:**
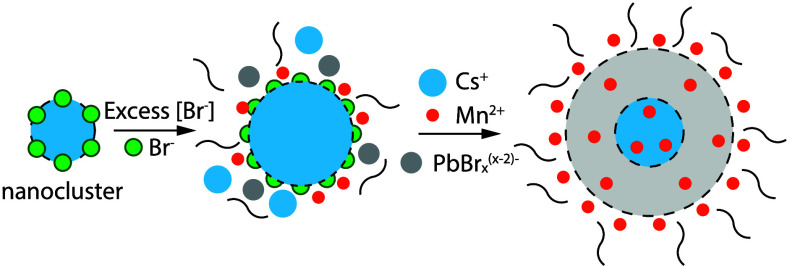
Diagram showing the suggested growth mechanism of Mn^2+^-doped CsPbBr_3_ QDs. Schemes are not in-scale.

To provide some experimental insights into the proposed doping mechanism, we have systematically investigated the effect of changes in synthesis conditions on the structural and optical properties of the obtained Mn^2+^-doped QDs (Table S1). First, the Mn­(Ac)_2_·4H_2_O and HBr reaction is crucial for achieving efficient doping. Reducing the amount of Mn­(Ac)_2_·4H_2_O or withdrawing the 230 °C heating process (Figure S26) will decrease the Mn^2+^ doping efficiency. This is consistent with the surface adsorption-driven mechanism of Mn^2+^ incorporation. Second, reducing the bromide concentration (HBr) will lead to low Mn^2+^ incorporation, which indicates the necessity of bromide-rich conditions. Third, increasing the Cs precursor concentration will not significantly compromise the Mn^2+^ doping and the degree of Cs deficiency in the Mn^2+^-doped QDs. This is expected in the proposed electrostatic surface adsorption model since Mn^2+^ possesses a higher charge density than Cs^+^. Additionally, reducing the Cs precursor concentration will only decrease the reaction yield of the synthesis but not the Mn^2+^ incorporation. This insensitivity to the amount of Cs precursors supports the Mn^2+^ surface adsorption model. Finally, the QD growth can be suppressed by loading additional Mn­(Ac)_2_·4H_2_O/HBr during synthesis. This allows for the synthesis of Mn^2+^-doped QDs with a size of only 3 nm (±0.3 nm) (Figure S27). This strongly suggests the efficient surface Mn^2+^ adsorption, which could cover the surface and suppress the QD growth if additional Mn^2+^ ions are introduced.

The Mn^2+^ surface adsorption model suggests that Mn^2+^ ions incorporated into lattices near the QD surface may experience a different chemical environment. Due to the lack of Cs^+^, increased lattice distortion is expected, which could affect the Mn coordination environment. This hypothesis can explain the above observation that some Mn^2+^ ions are less thermally stable than others, as well as the revelation that there are two different Mn^2+^ PL decay time constants. Given that the nonstoichiometric surface lattices can offer additional flexibility in Mn^2+^ incorporation, it should be noted that the possibility of Mn^2+^ doping through occupying Cs^+^ sites or even interstitial doping cannot be ruled out. In fact, in Figure 3c, the (110) XRD peak of doped QDs broadens and shifts toward smaller angles compared to that of undoped QDs. This suggests crystal structural changes, such as increased lattice inhomogeneity and lattice expansion induced by possible interstitial dopants.^62,63^ It is also worth noting that the interstitially doped Mn^2+^ can also exhibit the typical d-d PL emission.^63^ The potential Cs^+^-substitutional or interstitial Mn^2+^ doping can lead to a higher apparent dopant concentration estimated by elemental analysis. Finally, this doping strategy can also be applied to CsPbCl_3_ QDs. Although successful Mn^2+^ doping has been demonstrated in CsPbCl_3_ QDs, our method can dope CsPbCl_3_ QDs more efficiently, manifested as a significantly red-shifted Mn^2+^ PL band (from ∼609 to 650 nm) due to strong Mn–Mn coupling (Figure S28). In principle, the nonstoichiometric perovskite QDs can lift the barrier of the highly dynamic surface of perovskite QDs for a large variety of impurity doping.

## Conclusion

In conclusion, we have demonstrated that size-confined CsPbBr_3_ QDs can be efficiently doped with Mn^2+^. The Mn^2+^-doped QDs exhibit 90 ± 10% Mn^2+^ emission PLQY and extraordinary PL stability. These doped QDs are nonstoichiometric as they are Cs-deficient. Detailed structural characterization shows that the surface of the doped QDs is covered with a large concentration of Mn^2+^ that cannot be removed using traditional purification methods such as antisolvent precipitation and GPC column. A chemical redox reaction-based purification is developed to thoroughly remove the surface-adsorbed Mn^2+^, enabling the detailed quantification of lattice-incorporated Mn^2+^ in CsPbBr_3_ QDs. Using QDs with simultaneously high PLQY and quantum confinement, the exciton-to-dopant energy transfer rate was measured for the first time in Mn^2+^-doped CsPbBr_3_ QDs. The ET rate of ∼0.06 ns^–1^ suggests an intrinsic suppressed exchange interaction between the excitons and Mn^2+^ in CsPbBr_3_ compared to CsPbCl_3_. The nonstoichiometric QD facilitated surface adsorption promises facile and efficient doping and alloying cationic impurities into traditionally “almost undopable” CsPbBr_3_ QDs.

## Experimental Section

### Materials

The following chemicals were used as received: cesium carbonate (Cs_2_CO_3_, puratronic, 99.994% metals basis, Alfa Aesar), lead­(II) bromide (PbBr_2_, puratronic, 99.999% metals basis, Alfa Aesar), manganese acetate tetrahydrate (Mn­(CH_3_COO)_2_·4H_2_O, ACROS Organics), oleylamine (OAm, technical grade, 70%, Sigma-Aldrich), oleic acid (OA, technical grade, 90%, Sigma-Aldrich), 1-octadecene (ODE, technical grade, 90%, Sigma-Aldrich), hydrobromic acid (HBr, 48 wt % in water, Acros), hydrogen peroxide solutions (H_2_O_2_, 30 wt., ACS grade, Sigma-Aldrich), nitric acid (HNO_3_, 67–70% w/w, VWR Chemicals), rhodamine 6G perchlorate (99%, Aldrich Chemistry), acetone (certified ACS, Fisher), hexanes (HPLC grade, Millipore), toluene (ACS grade, Fisher), methyl acetate (ReagentPlus, 99%, Sigma-Aldrich) acetonitrile (C_2_H_3_N, >99.9%, Sigma-Aldrich) and tetrabutylammonium hexafluorophosphate (TBAPF_6_, Sigma-Aldrich). Bio-Beads S-X1 GPC medium was sourced from Bio-Rad Laboratories.

### Cs-Precursor Preparation

The Cs-oleate precursor was prepared by dissolving 300 mg of Cs_2_CO_3_ in a mixture of 1.2 mL of OA and 3.2 mL of ODE in a 50 mL three-necked round-bottom flask. This mixture was degassed under a vacuum at room temperature for 3–5 min while stirring vigorously. The mixture was then heated to 130 °C under vacuum. Following this, the flask was filled with nitrogen and cooled to 120 °C for subsequent use.

### Mn^2+^-Doped CsPbBr_3_ QD Synthesis

60 mg of PbBr_2_ (0.16 mmol) and 255 mg of Mn (CH_3_COO)_2_·4H_2_O (Mn­(Ac)_2_·4H_2_O, 1.04 mmol) were added in a three-neck round-bottom flask, followed with 1 mL of OA, 1 mL of OAm, 5 mL of ODE, and 0.5 mL of HBr. The mixture was heated at 150 °C under vacuum for 40 min. The flask was then filled with nitrogen, and an additional 1 mL of dried OA and 1 mL of dried OAm were injected to solubilize the unreacted solids. To obtain dried OA and OAm, both ligands were dried under vacuum in separate flasks at 150 °C for 15 min. The solution was next heated to 200 °C for 40 min, after which the temperature was increased to 230 °C and held for 5–10 min until stabilized. Then, the reaction mixture was allowed to decrease to 173 °C, and 0.7 mL of the Cs-precursor solution (containing 0.3 mmol CsOA) was injected. Following a short reaction time of ∼5 s, the reaction was rapidly quenched with an ice bath. The crude solution was centrifuged at 7800 rpm for 7 min, and the precipitate was discarded. The QDs were precipitated from supernatant by adding acetone in a 4:1 volume ratio, followed by centrifugation at 7800 rpm for 7 min. The supernatant was discarded, and the precipitate was then resuspended in toluene. QDs with various Mn^2+^ PLQYs can be achieved by varying the amount of Mn­(CH_3_COO)_2_·4H_2_O and injection temperature; details can be found in Table S1. Undoped QDs were prepared by adopting a previously reported method.^35^ For synthesizing QDs with Mn^2+^ PLQY above ∼50%, the doping could be tuned by simply changing the amount of Mn­(Ac)_2_·4H_2_O/HBr precursors (up to 255 mg and 0.5 mL, respectively) without adjusting other conditions. For synthesizing QDs with lower Mn^2+^ PLQYs, the injection temperature was increased (up to 200 °C), and the amount of Mn­(Ac)_2_·4H_2_O/HBr precursors was decreased.

### Antisolvent Purification

Mn^2+^-doped QDs colloid was then purified by adding methyl acetate in a 3:1 volume ratio and centrifuged at 7800 rpm for 7 min to reprecipitate QDs. The collected precipitate was redispersed in hexanes or toluene for storage and further characterizations. QDs with various Mn^2+^ PLQYs can be achieved by varying the amount of Mn­(CH_3_COO)_2_·4H_2_O and injection temperature; details can be found in Table S1. Undoped QDs were prepared by adopting a previously reported method.^35^

### GPC Purification of Mn^2+^-Doped QDs

Mn^2+^-doped QDs were purified by GPC using the method reported by B. Greytak et al.^45^ Briefly, 4–5 g of Bio-Beads were washed three times and soaked overnight using toluene before use. Next, ∼5 mL of toluene was added to a glass column, which was carefully blocked with glass wool to prevent any bead leakage. The soaked Bio-Beads were then transferred to the column to reach a bed height of 10 cm. Once the column was fully packed, it was thoroughly rinsed with toluene until no free polystyrene was detected in the eluent, as confirmed by UV–vis absorption and fluorescence. Then, 1 mL of QD colloid was carefully loaded into the GPC column. The collected QDs were loaded into another GPC column after chemical purification for further purification iterations. The eluted QDs were collected and stored for elemental analysis and spectroscopic measurements.

### Chemical Purification

20 μL of H_2_O_2_ solution (30 wt %) was added to the Mn^2+^-doped CsPbBr_3_ QD toluene solution. The solutions were then shaken to initiate Mn^2+^ oxidation. The Mn^2+^-doped CsPbBr_3_ QD solution will turn a dark brown color and start bubbling. The organic phase was collected, 20 μL of HBr was added, and the container was shaken for 15 s. After the organic phase became clear, the solution was centrifuged to accelerate phase separation, and the organic phase (QD colloid) was collected for further analysis. It is worth noting that the Mn^2+^ PL stability can vary depending on the relative amount of QDs and H_2_O_2_ solutions.

### Elemental Analyses

The composition of QDs was determined using ICP-MS (Agilent 7850). The QD solutions using various purification methods were digested in concentrated nitric acid for ICP-MS analyses. The Br content in the halide perovskite was determined by STEM and EDS using Titan Themis 300 S/TEM. QD samples for XPS analysis were prepared by drop-casting the purified QD solution onto clean silicon substrates. An Omicron DAR 400 equipped with a CN10 charge neutralizer was used to collect the XPS data.

### Structural Characterizations

Electron microscope images of all nanocrystals were obtained using a Titan Themis 300 TEM microscope operated at 300 kV. DLS analysis of CsPbBr_3_ NCs and Mn^2+^-doped CsPbBr_3_ QDs suspended in hexanes was recorded using a pUNk v1.0.0.3 DLS system equipped with a red laser (660 nm). The light scattering intensity was measured as a photon count rate in units of kilocounts per second (kcps). To account for varying scattered light intensities from nanoparticles of different sizes, the instrument automatically adjusted the incident laser beam power to achieve an optimal photon count rate. A built-in attenuator was used to set the laser power to specific levels as required. ζ-potential measurements were performed using the Anton Paar Litesizer 500. A suspension of Mn^2+^-doped or undoped CsPbBr_3_ nanocrystals (NCs) in toluene was introduced into a Univette equipped with a quartz cuvette containing 700 μL acetonitrile and 5–20 μL of 10 mM tetrabutylammonium hexafluorophosphate (TBAPF_6_) in acetonitrile as the supporting electrolyte. XRD patterns for CsPbBr_3_ NCs and Mn^2+^-doped CsPbBr_3_ NCs were collected on a Rigaku SmartLab X-ray diffractometer with a Cu–Kα source. The samples were prepared by drop-casting purified NCs onto a zero-background sample holder to ensure precise detection of XRD signals. Measurements were performed at room temperature with scans recorded in the 10–40° (2θ) range. Data analysis was conducted using Rigaku’s PDXL2 software package.

### NMR Experiments

The ^1^H and ^133^Cs NMR spectra were acquired at a probe temperature of 20 °C on a 500 MHz JEOL ECZL NMR instrument (operating at 500.16 MHz for proton and 65.60 MHz for cesium) equipped with a 5 mm gradient inverse broadband Royal probe. The 1H NMR spectrum was recorded with single pulse excitation, a spectral width of 7508 Hz, and a pulse width of 3.95 μs (45° flip angle). A scan number of 8 transients and a repetition rate of 4.19 s (3.19 s for the acquisition time and 1 s for the relaxation delay) were used. The raw data (FIDs) for protons were processed without any apodization function prior to Fourier transformation. The ^133^Cs NMR spectrum was recorded with a spectral width of 19723.9 Hz with 40K data points and a pulse width of 7.02 μs (45° flip angle). A scan number of 4096 transients and a repetition rate of 3.66 s (1.66 s for the acquisition time and 2 s for the relaxation delay) were used. Exponential weighting with a line-broadening function of 25 Hz was applied before the Fourier transformation. ^133^Cs is the only naturally occurring stable isotope of cesium with a nonzero nuclear spin (I) of 7/2. Since it has a very small quadrupole moment (−3 × 10^–3^) (eQ) × 10^–28^ C m^–2^, a high natural abundance (100%), and a low magnetogyric ratio (3.5277 × 10^7^ rad T^–1^ s^–1^) and has an excellent receptivity relative to the value of 269 for ^13^C. ^1^H and ^133^C NMR chemical shifts for protons are reported in parts per million (ppm) and are referenced to residual protons in the solvent peak CHCl_3_ at 7.26 ppm. ^133^Cs NMR chemical shifts are referenced using the cesium peak from Cs_2_CO_3_ at 0.0 ppm as the external standard.

### EPR Experiments

The EPR spectra were acquired at room temperature using a commercial Bruker Biospin EMX spectrometer operating at a frequency of 9.51 GHz. The Mn^2+^-doped CsPbBr_3_ NCs were suspended in an EPR-inactive hexane or toluene solvent, and a few milliliters of the solution were put in 4 mm OD low-loss quartz tubes. The tubes were then inserted in the middle of the cylindrical microwave cavity for the EPR measurements. Typical microwave powers of 1–2 mW with 3 G modulation amplitude and 100 kHz field modulation were employed for these experiments. The Zeeman splitting g-values were calibrated using a DPPH (2,2-diphenyl-1-picrylhydrazyl) standard. The magnetic field resonance values and FHWM line widths were determined from fits to the spectra using Lorentzian line shapes (i.e., the first derivative of the Lorentzian-shaped microwave absorption curves).

Low-temperature EPR spectra were recorded with a Bruker/ColdEdge ESR900 WaveGuide cryostat, operating at a frequency of 9.35 GHz. This setup included a liquid helium flow system, allowing temperature control between 3 and 300 K. The Mn^2+^-doped CsPbBr_3_ NCs were dissolved in an EPR-inactive toluene solvent, with several milliliters of the mixture placed into 4 mm OD thin-wall Suprasil EPR tubes. The experiments were conducted using standard microwave power levels ranging from 0.2 to 2 mW, featuring a modulation amplitude of 3 G and a field modulation frequency of 100 kHz. Calibration of the Zeeman splitting g-values was performed using BDPA (α,γ-bisdiphenylene-β-phenylallyl) standard.

### Optical Spectroscopy Measurements

An Ocean Insight Maya 2000 spectrometer was used to record absorption and PL spectra. A 385 nm LED was used to excite the samples for PL measurements. The PLQY of QDs was measured with respect to rhodamine 6G standard (PLQY 95%) as well as a reference QD sample (Mn^2+^-doped CdS/ZnS, PLQY 60.4% (Figure S2b), both excited with OBIS LX SF 405 nm continuous-wave laser (Coherent). QDs and Rhodamine 6G were suspended/dissolved in hexane and ethanol, respectively, and the difference in solvent refractive indices was accounted for when calculating the PLQY. Absolute PLQY was measured using an integrating sphere (Labsphere) coupled with a computer-controlled spectrometer (Ocean Optics QE Pro). The light sources used were a 405 nm laser diode (LDM405, Thorlabs, 4.0 mW) and a 415 nm fiber-coupled LED excitation source (M415F3, Thorlabs, 14.4 mW). PLE measurements were performed on colloidally suspended, highly Mn^2+^-doped CsPbBr_3_ nanocrystals in hexanes, using a quartz cuvette as the sample holder. The measurements were conducted with a HORIBA Jobin Yvon Fluorolog-3 spectrofluorometer equipped with a xenon lamp. Data were collected using the two-curve method over a wavelength range of 250–750 nm.

Pump–probe TA measurements were conducted at room temperature using a HELIOS TA spectrometer from Ultrafast Systems. The 405 nm, 2 kHz output from an Apollo-Y Optical Parametric Amplifier (OPA) served as the pump beam, with a fluence of ∼18.8 μJ/cm^2^. The probe beam, with a white-light spectrum, was generated in a sapphire crystal within the HELIOS spectrometer by focusing the 1064 nm, 2 kHz output from a Hyperion femtosecond amplified laser. Both the pump and probe pulses had a duration of approximately 350 fs.

### Time-Resolved Photoluminescence (TRPL) Spectroscopy

The exciton PL lifetimes were measured using a time-correlated single-photon counting technique. The colloidal sample in a cuvette was excited with a 405 nm pulsed laser (Picoquant LDH-D-C-405) driven by a Picoquant Sepia PDL828 module at a 5 MHz repetition rate. The PL emission from the Mn^2+^-doped CsPbBr_3_ NCs was collected with an achromatic lens, sent through a set of long-pass (425 nm, Edmund Optics #84-742) and bandpass (466 ± 20 nm, Edmund Optics #86-352) filters to remove the scattered laser and the Mn^2+^ emission, and detected using a single-photon avalanche photodiode (Hamamatsu C11202-100). The photon arrival time was recorded using a Picoquant HydraHarp 400 correlator. The Mn^2+^ PL lifetimes were measured by exciting the sample using the 375 nm, 80 MHz output of a pulsed laser (Beckl & Hickl). The average excitation power was 30 μW, focused to a beam diameter of around 200 μm. PL was collected in free-space, backscattering geometry, and spectrally resolved with an HRS-300 Acton Spectrometer coupled to a Teledyne PIXIS400 CCD camera. The temporal resolution of the PL decay was achieved using a Time-Correlated Single Photon Counting (TCSPC) system by Becker & Hickl, comprising an IDQ-id100 fast avalanche photodiode and an SPN 130, capable of simultaneously measuring both nanosecond (fluorescence) and millisecond (phosphorescence) decay components. The phosphorescence decay time is measured in a triggered accumulation multichannel scaler (TA-MCS) mode, where the high-frequency laser output is modulated at lower frequencies.^64^ For an in-depth description of the electronics behind the TA-MCS mode, please refer to the Beckl & Hickl available online free of charge.

## Supplementary Material



## Notes

Additional optical measurements (UV–vis, PL, PLQY, TRPL, TA), elemental analyses (EDS, ICP-MS, XPS), STEM imaging, NMR, EPR, synthesis conditions, additional photographs of QD samples, and TRPL fitting parameters. (PDF)
