# Supplementary material for: Efficient Mn2+ Doping in Non-Stoichiometric Cesium Lead Bromide Perovskite Quantum Dots
Source: J Am Chem Soc. 2025 Sep 15;147(38):35069–80. doi: 10.1021/jacs.5c12086 (PMC12464970; doi:10.1021/jacs.5c12086)
Supplement: Supplementary file 1 [file ja5c12086_si_001.pdf]

Supporting Information

Efficient Mn^{2+} Doping in Non-Stoichiometric Cesium Lead Bromide Perovskite Quantum Dots

Lamia Hidayatova[1], Chenjia Mi[1], Novruz G. Akhmedov[1], Yuan Liu,[2] Arjumand K. Shafiq[1], Hadi Afshari[3], Nishya Mohamed-Raseek[1], Dilruba A. Popy[1], Sisi Xiang[4,] Yi-Chen Chen[2], Bayram Saparov[1], John W Peters[1], Dmitri V. Talapin[2,5], Bin Chen[6], Madalina Furis[3,7], Evan R. Glaser[8], and Yitong Dong[1,7*]

[1]*Department of Chemistry and Biochemistry, The University of Oklahoma, Norman, Oklahoma 73019, USA*

[2]Department of Chemistry, James Franck Institute, and Pritzker School of Molecular Engineering, University of Chicago, Chicago, Illinois 60637, United States

[3]*Homer L. Dodge Department of Physics and Astronomy, The University of Oklahoma, Norman, Oklahoma 73019, USA*

[4]*Department of Materials Science and Engineering, Texas A&M University, College Station, Texas 77843, USA*

[5]*Center for Nanoscale Materials, Argonne National Laboratory, Argonne, Illinois 60439, United States*

[6]*Department of Chemistry, Northwestern University, Evanston, Illinois 60208, USA*

[7]*Center for Quantum Research and Technology, The University of Oklahoma, Norman, Oklahoma 73019, USA*

[8]*U.S. Naval Research Laboratory, Washington D.C. 20375, USA*

**Corresponding author*

Email: Yitong.Dong-1@ou.edu

Table of Contents

Table S1 ..... *4*

Supporting Information Note 1 ..... *4*

Figure S1 ..... *5*

Figure S2 ..... *5*

Figure S3 ..... *6*

Figure S4 ..... *6*

Figure S5 ..... *6*

Figure S6 ..... *7*

Table S2 ..... *7*

Supporting Information Note 2 ..... *7*

Figure S7 ..... *8*

Figure S8 ..... *9*

Figure S9 ..... *9*

Figure S10 ..... *10*

Figure S11 ..... *10*

Figure S12 ..... *10*

Figure S13 ..... *11*

Figure S14 ..... *11*

Figure S15 ..... *11*

Figure S16 ..... *12*

Figure S17 ..... *12*

Figure S18 ..... *12*

Figure S19 ..... *13*

Supporting Information Note 3 ..... *13*

Figure S20 ..... *13*

Supporting Information Note 4 ..... *13*

Figure S21 14
Figure S22 14
Figure S23 15
Table S3 15
Figure S24 16
Figure S25 16
Supporting Information Note 5 16
Figure S26 17
Figure S27 17
Figure S28 18
References 19

Table S1. Correlation between synthesis conditions and properties of Mn^{2+}-doped $CsPbBr_3$ QDs. Sample A represents the synthesis condition optimized for the maximum ratio of Mn^{2+} PL versus exciton PL. The changes in the synthesis conditions are color-coded. In all syntheses, 0.163 mmol of $PbBr_2$ was added.

QD Sample	$Mn(Ac)_2 \cdot 4H_2O$ (mmol)	HBr (mmol)	HBr/Mn molar ratio	Cs-oleate (mmol)	Reaction T (°C)	Estimated Size (nm)	Mn^{2+} PLQY (%)	Cs/Pb atomic ratio
A	1.04	4.42	4.25	0.29	173	~4	93	~0.60
B	0.163	0.88	5.40	0.29	173	~5	4.3	0.72
C	1.04	4.42	4.25	0.29	188	4-5	50	N/A
D	0.685	2.83	4.12	0.29	185	4-5	40	0.61
E	1.04	4.42	4.25	0.84	173	~4	64	0.60
F*	1.04	4.42	4.25	0.17	173	~3	79	N/A
G**	0.641	4.42	6.90	0.29	173	4-5	52	N/A
H**	1.04	0.88	0.85	0.29	173	N/A	10	N/A
I	1.16	4.68	4.10	0.29	173	~3	61	N/A

*The chemical yield of this QD sample was very low.
**The Mn^{2+} PL emission from these QDs was unstable and decreased over time

Supporting Information Note 1: In summary, the amount of Cs precursors added remains unchanged across different Mn^{2+}-doped QDs studied in **Figures 1,2,3, and 4**, and the doping efficiency/Cs deficiency is regulated by adjusting the amount of Mn precursors ($Mn(Ac)_2 \cdot 4H_2O$/HBr). As shown in **Table S1**, deliberately increasing the amount of injected Cs precursors causes a slight decrease in the Mn^{2+} PLQY (*Sample E*). However, the Cs/Pb ratio stays very low, indicating the stoichiometry is not sensitive to additional Cs precursors. Reducing the amount of Cs precursors (*Sample F*) results in QDs with similar optical properties to those produced using the original synthesis conditions (*Sample A*), but with a significantly lower reaction yield.

We then varied the reaction temperature and the amount of Mn precursors. Reducing the $Mn(Ac)_2 \cdot 4H_2O$/HBr precursor amount results in a very low Mn^{2+} PLQY (*Sample B*), indicating low Mn^{2+} incorporation. This is expected because Mn^{2+} was introduced in our doping process by reacting $Mn(Ac)_2 \cdot 4H_2O$ with HBr under heat and vacuum. We also observed that increasing the reaction temperature led to larger QD sizes (still strongly quantum confined) (*Samples C and D*). The high temperature may have negatively affected the Mn^{2+} surface adsorption, therefore resulting in a slightly reduced Mn^{2+} PLQY. Precipitates from the crude solution after centrifuging were collected for samples with an injection temperature above 185 °C.

Following this, we have independently varied the additions of HBr and the $Mn(Ac)_2 \cdot 4H_2O$. As shown in **Table S1**, reducing only Mn will decrease PLQY, but not significantly

(*Sample G*). However, the excess acid in the system compromised the stability of the QDs, rendering them unreliable for further characterization. On the other hand, reducing only the quantity of HBr will significantly lower the doping efficiency (*Sample H*), as $Mn(Ac)_2{\cdot}4H_2O$ needs to react with HBr to become solubilized in the reaction mixture. Finally, increasing $Mn(Ac)_2{\cdot}4H_2O$ and HBr together will lead to even smaller-sized (~3 nm) Mn^{2+}-doped QDs (*Sample I*). We attributed this to a tightly physiosorbed Mn^{2+} compound on the surface during QD growth in our proposed model, which has prevented very small QDs from further growth. We have added these discussions to the revised manuscript and provided the details of syntheses in the **Experimental Section**.

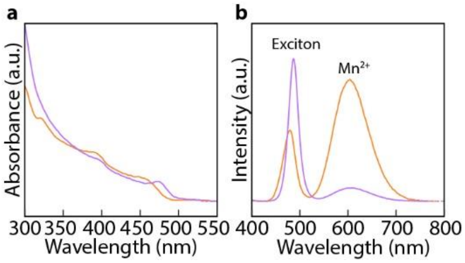

Figure S1. (a) Absorption and (b) photoluminescence (PL) spectra of Mn^{2+}-doped $CsPbBr_3$ QDs with different Mn^{2+} PLQYs (~67% (orange) and ~9% (purple)). Doping with more Mn^{2+} blueshifts the exciton PL peak.

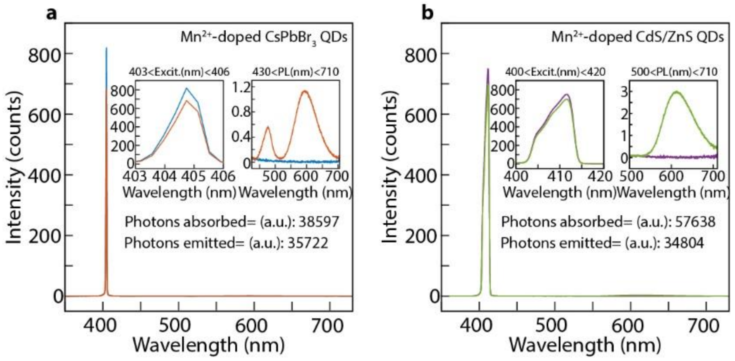

Figure S2. Absolute Photoluminescence Quantum Yield (PLQY) measurement of (a) Mn^{2+}-doped $CsPbBr_3$ (orange, PLQY=92.5%), (b) Mn^{2+}-doped CdS/ZnS QDs (green, PLQY=60.4%) using an integration sphere. Blanks (toluene only) are marked in (a) blue and (b) purple.

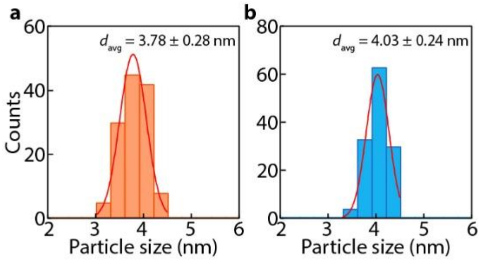

Figure S3. QD size distribution histograms derived from High-angle Annular Dark-field Scanning Transmission Electron Microscopy (HAADF-STEM) images of (a) Mn^{2+}-doped and (b) undoped $CsPbBr_3$ quantum dots (QDs).

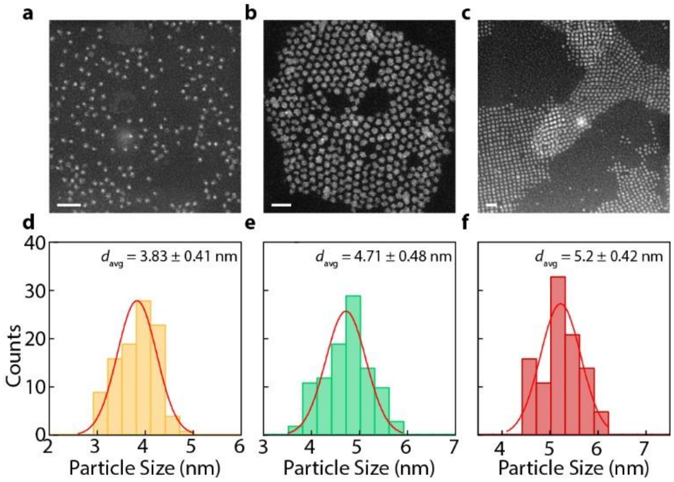

Figure S4. (a – c) HAADF-STEM images and (d – f) their corresponding size distribution histograms with Gaussian fitting curves (red lines) for Mn^{2+}-doped $CsPbBr_3$ QDs. Scale bars of HAADF-STEM images are 20 nm.

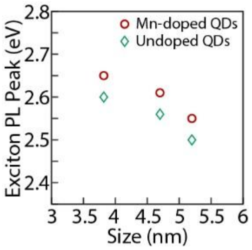

Figure S5. Exciton PL peak position (red circles) for Mn^{2+}-doped $CsPbBr_3$ QDs (measured) and the estimated exciton PL position (green diamonds) for undoped QDs

calculated from the sizing curve.[1] The deviation between these two indicates the effect of Mn^{2+} doping on bandgap behavior.

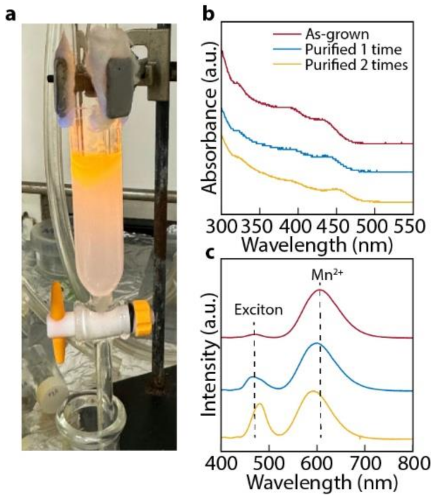

Figure S6. (a) Photograph of GPC purification of Mn^{2+}-doped $CsPbBr_3$ QDs. (b) Absorption and (c) PL spectra of Mn^{2+}-doped QDs before and after purification using two separate columns. The exciton red shift observed in the Mn^{2+}-doped nanocrystals can be attributed to a small amount of dopant loss due to surface degradation that may have been induced during purification. The potential dopant loss due to surface damage is also reflected in the PL spectra as a slight blue shift in the Mn^{2+} PL peak. Note that we have only applied one-time GPC purification before and after chemical purification, as mentioned later in the text, to minimize the damage and dopant loss.

Table S2. Cation compositions of a Mn^{2+}-doped QD sample after purification using two separate GPC columns.

	Cs/Pb	Mn/Pb
As-grown	0.60	30
Purified GPC 1	0.59	29
Purified GPC 2	0.62	23

Supporting Information Note 2: Chemical stoichiometry of undoped strongly confined $CsPbBr_3$ QDs.

To clarify the determination of Cs deficiency, we have used a reported QD model with a composition of $CsPbBr_3(PbBr_2)(ABr)$ as a reference QD stoichiometry.[2] In this model, all surface Cs ions are replaced by cationic ligands such as ammonium, and all surface lead is fully covered by bromide. Our bromide-rich synthesis conditions will likely generate QDs with AX terminations. The general chemical stoichiometry for cubic $CsPbBr_3$ QDs containing (n>2) lattices from edge to edge is $Cs_{n^3}Pb_{(n+1)^3}Br_{3(n+1)^2(n+2)}$. When the QD size is small, the stoichiometry can significantly deviate from the bulk (1:1:3) and will approach the bulk as the size increases. Given that our QD size is 4-5 nm, we have adopted the reference stoichiometry of undoped QDs with n=7-8, which gives us a chemical stoichiometry of $Cs_{0.67-0.7}PbBr_{3.3-3.4}$. Such a reference stoichiometry is verified in our study: elemental analyses (ICP-MS and TEM-EDS) show an average chemical composition of $Cs_{0.73-0.9}PbBr_{3.2-4}$.

In this study, the Cs/Pb ratio in Mn^{2+}-doped QDs is consistently reduced to approximately 0.6 (as low as 0.55) due to Mn^{2+} doping. With Mn^{2+} substitutional doping, there are fewer Pb^{2+} atoms in the doped QDs; therefore, the actual percentage of Cs^+ loss (estimated by using a reference Cs/Pb ratio of 0.7) will be higher, reaching about 50% for Mn^{2+}-doped QDs with a doping concentration of around 40%.

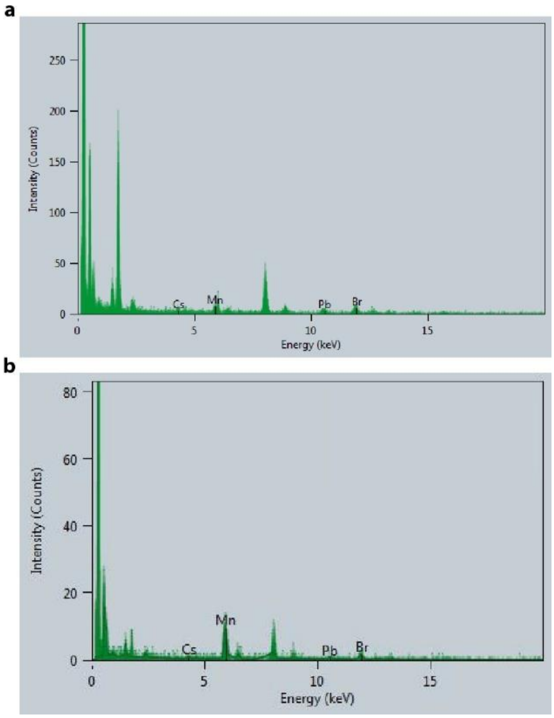

Figure S7. Energy Dispersive X-ray Spectroscopy (EDS) spectra of Mn^{2+}-doped $CsPbBr_3$ QDs with (a) 24% and (b) 44% Mn^{2+} PLQY.

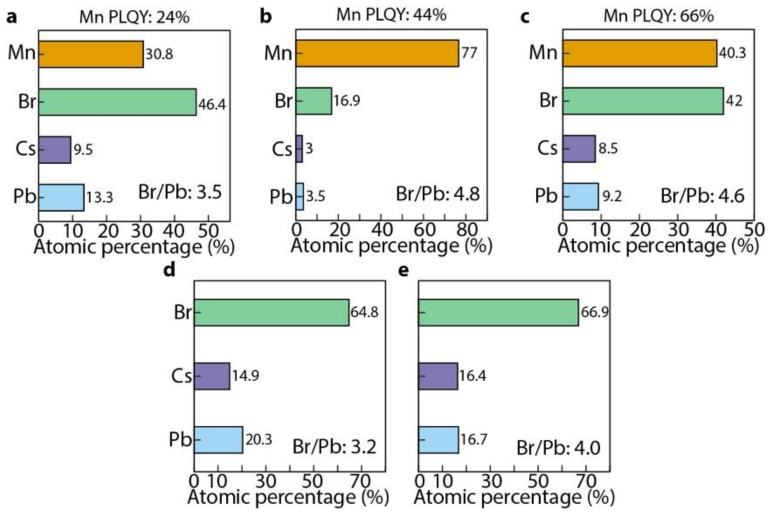

Figure S8. Quantified atomic percentages of (a-c) Mn^{2+}-doped $CsPbBr_3$ QDs and (d, e) undoped. $CsPbBr_3$ QDs are synthesized using the reported method.[3] Sample (c) has been purified using the chemical method; however, the presence of organic ligands and possible residual Mn^{2+} residues reduced the accuracy of the analysis. Therefore, the composition results from EDS measurements have not been used to quantify the stoichiometry in this work.

Slightly higher bromide-rich compositions are observed in Mn^{2+}-doped $CsPbBr_3$ QDs compared to undoped QDs, consistent with their synthesis in a more anion-rich environment.

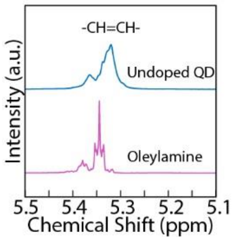

Figure S9. ^1H NMR spectra of technical grade oleylamine (pink) and undoped $CsPbBr_3$ QDs (blue) in chloroform-d ($CDCl_3$), showing the signal of the alkenyl protons on oleylamine/oleylammonium. The peak broadening indicates the attachment (and passivation) of the oleylammonium bromide on the QDs.

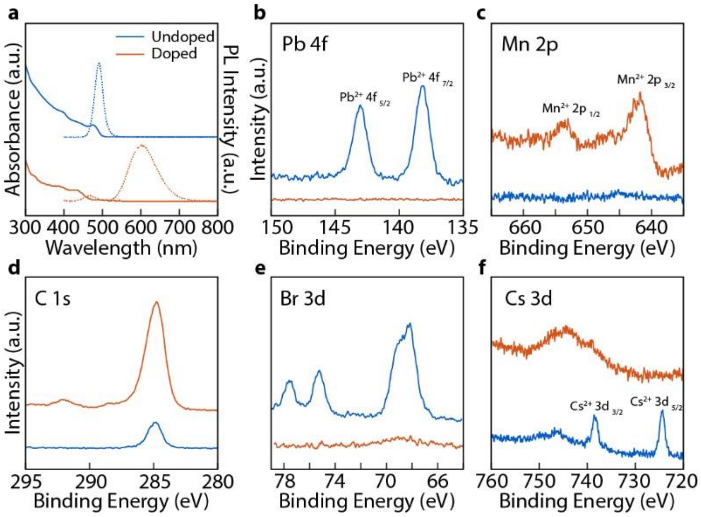

Figure S10. (a) Absorption and PL spectra of undoped and Mn^{2+}-doped $CsPbBr_3$ QDs. High-resolution XPS spectra of (b) Pb 4f, (c) Mn 2p, (d) C 1s, (e) Br 3d, and (f) Cs 3d of undoped and Mn^{2+}-doped $CsPbBr_3$ QDs. The samples were prepared by drop-casting nanocrystals onto silica substrates. Strong characteristic Pb 4f and Mn 2p XPS peaks were observed for undoped and Mn^{2+}-doped nanocrystals, respectively. However, a high concentration of Mn-oleates may have screened the characteristic Pb, Br, and C photoelectrons in the doped nanocrystals. The spectra are plotted without rescaling.

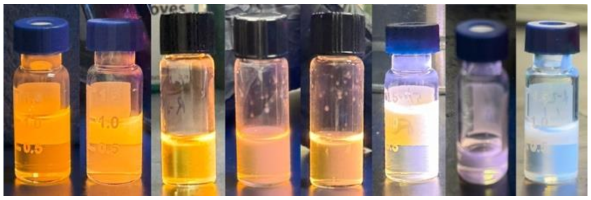

Figure S11. Additional photographs of Mn^{2+}-doped $CsPbBr_3$ QDs under UV illumination.

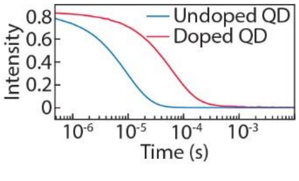

Figure S12. Dynamic light scattering (DLS) correlation functions for Mn^{2+}-doped (red curve) and undoped (blue curve) $CsPbBr_3$ QDs.

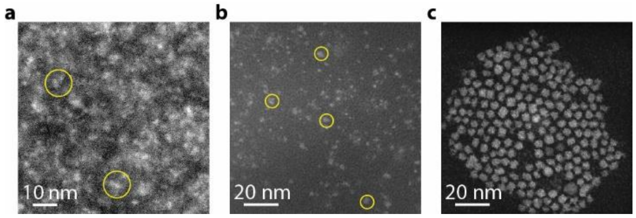

Figure S13. HAADF-STEM images of (a) antisolvent-resuspension, (b) GPC-chemical and (c) GPC-chemical-GPC purified aggregated Mn^{2+}-doped $CsPbBr_3$ QDs.

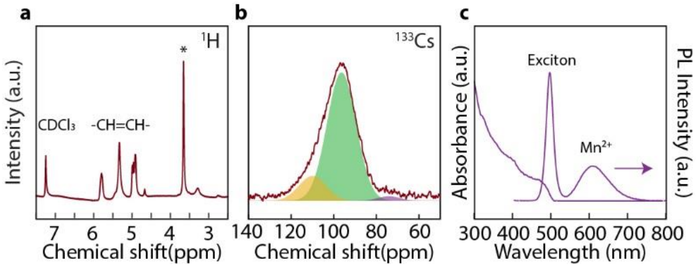

Figure S14. (a) ^{1}H and (b) ^{133}Cs NMR spectra of doped QDs with lower Mn^{2+} PLQY efficiency. The ^{133}Cs NMR spectrum is best fit with three components: core (green), intermediate (purple), and surface (yellow) Cs species. (c) Absorption and PL spectra of Mn^{2+}-doped QDs with lower Mn^{2+} PLQY.

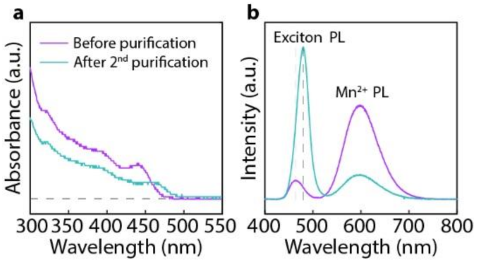

Figure S15. (a) Absorption and PL (b) spectra for Mn^{2+}-doped $CsPbBr_3$ QDs before and after two purification cycles.

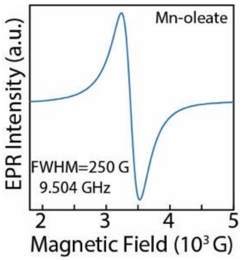

Figure S16. The electron paramagnetic resonance (EPR) of a Mn-oleate solution.

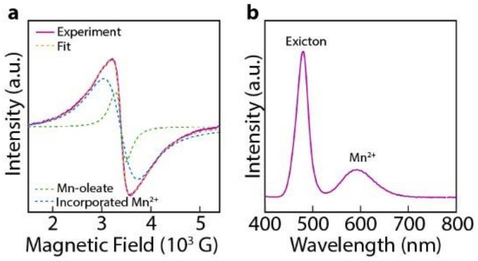

Figure S17. (a) EPR spectrum of Mn^{2+}-doped $CsPbBr_3$ QDs with ~20% Mn^{2+} PLQY includes the experimentally obtained spectrum (purple), the fitted spectrum (yellow), the contribution from lattice-incorporated Mn^{2+} (blue), and surface-adsorbed Mn^{2+} (green). (b) PL spectrum of the sample used for EPR measurement.

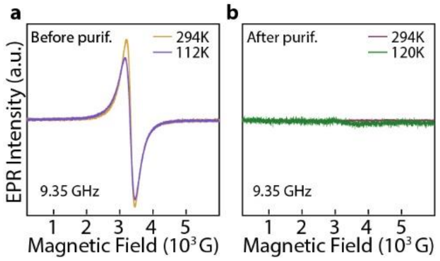

Figure S18. Temperature-dependent EPR spectra. (a) EPR spectra of Mn^{2+}-doped $CsPbBr_3$ QDs before chemical purification at 294 K and 112 K. (b) EPR spectra of Mn^{2+}-doped $CsPbBr_3$ QDs after GPC-chemical-GPC purification at 294 K and 120 K.

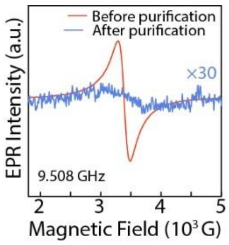

Figure S19. EPR spectra of Mn^{2+}-doped $CsPbBr_3$ QDs used for spin concentration quantification and ICP-MS analysis before (orange) and after (blue) GPC-chemical-GPC purification.

Supporting Information Note 3: The spin concentration of the QD sample before GPC-chemical-GPC purification was quantified as 2.9×10^{20} spins/mL, corresponding to 0.096 M Mn, considering each Mn^{2+} ion has 5 unpaired electrons. ICP-MS analysis was performed on the same sample (details provided in the Experimental Section) to determine the concentration of Mn^{2+} ions (including both lattice incorporated and surface adsorbed Mn), resulting in a Mn concentration of 158.26 ppb (2.87 μM), corresponding to 0.086 M of Mn^{2+} in the original QD sample, which is in good agreement with the EPR results.

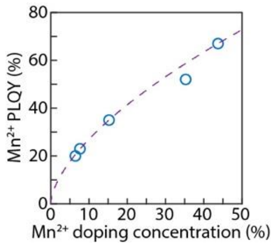

Figure S20. Correlation between Mn^{2+} PLQY and Mn^{2+} doping concentration of Mn^{2+}-doped $CsPbBr_3$ QDs. All samples have been purified using the newly developed GPC-chemical-GPC approaches to ensure the thorough removal of surface-physiosorbed Mn^{2+}. The dotted line is a sublinear (power law) fit that passes through the origin point. The fitting function is used to estimate the doping concentration of any Mn^{2+}-doped QDs with known Mn^{2+} PLQYs.

Supporting Information Note 4: Application of Vegard's Law for estimation of Mn^{2+}-dopant concentration based on XRD.

Vegard's Law[4] describes the linear relationship between an alloy's characteristics and the molar proportion of the component materials. It claims that an alloy's lattice constant can be approximated as a weighted average of the lattice constants of its pure components. Following Bailey and Nie (2003)[5], we employ the mathematical expression $E_{alloy}=xE_A+(1-x)E_B$, where E_A and E_B represent the contributions of pure components A and B, with x reflecting the fraction of one component in the mixture. In this case, we

apply Vegard's Law applies to the solid solutions of $CsPbBr_3$ and $CsMnBr_3$ nanocrystals (NCs), predicting that the lattice constant of the mixed system follows a similar linear relationship. The molar percentage of $CsMnBr_3$ determines the degree of deviation from the pure $CsPbBr_3$ lattice parameter[6].

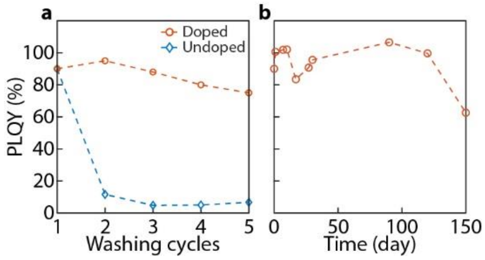

Figure S21. (a) PLQYs of Mn^{2+}-doped and undoped QDs before and after 5 iterations of precipitation-resuspension purification cycles using methyl acetate as the anti-solvent. (b) PLQY stability of doped QDs over 120 days shows modest luminescence intensity changes, indicating good long-term stability.

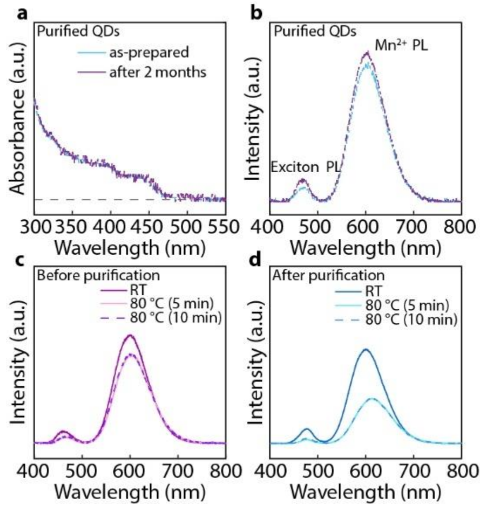

Figure S22. (a) Absorption and (b) PL spectra of chemically purified Mn^{2+}-doped $CsPbBr_3$ QDs measured on the first day of purification (blue) and after 2 months of storage (purple). PL spectra of Mn^{2+}-doped $CsPbBr_3$ QDs (c) before purification and (d) after GPC-chemical-GPC purification at room temperature (RT) and 80 °C.

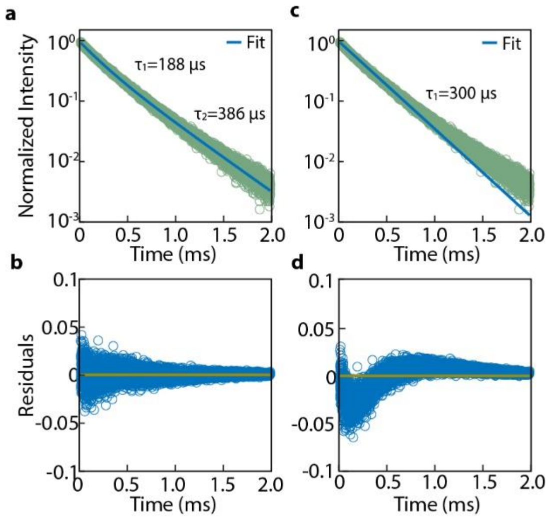

Figure S23. PL intensity decay curve of Mn^{2+}-doped $CsPbBr_3$ QDs fitted using (a) bi-exponential and (c) mono-exponential decay functions, with corresponding (b) and (d) residual plots. The residuals from a mono-exponential fit show a structural feature, indicating a less accurate fit compared to the biexponential fit. All fitting parameters are listed in **Table S3**.

Table S3. Fitting parameters of the Mn^{2+} PL intensity decay curve of Mn^{2+}-doped $CsPbBr_3$ QDs fitted using bi-exponential and mono-exponential decay functions.

	a_1 (%)	a_2 (%)	τ_1 (µs)	τ_2 (µs)	R^2	RMSE
Bi-exponential fit	58.26	40.90	188	386	1.000	0.0015
Mono-exponential fit	100	-	300	-	0.9704	0.0378

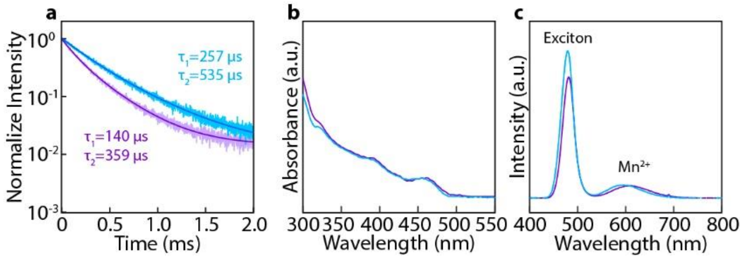

Figure S24. (a) Phosphorescence decay curves measured at the peak wavelength (600 nm) of the Mn^{2+} emission band, (b) absorption, and (c) PL spectra of two Mn^{2+}-doped QD samples with low Mn^{2+} doping concentrations. The blue-shifted Mn^{2+} PL peak position in (c) implies weaker Mn-Mn coupling, which is in good agreement with the longer PL lifetimes observed in (a).

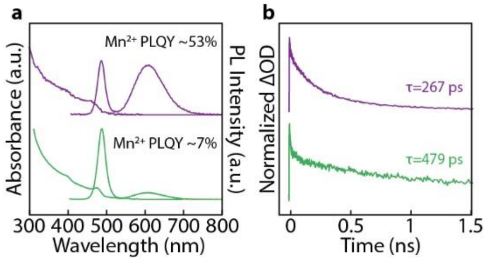

Figure S25. (a) Absorbance and PL spectra and (b) bleach recovery dynamics extracted from the TA spectra of Mn^{2+}-doped $CsPbBr_3$ QDs with varying Mn^{2+} PLQYs (~53% (purple) and ~7% (green)). The pump (405 nm) fluence was maintained at ~18.8 μJ/cm² per pulse.

Supporting Information Note 5. TA spectra were measured using Mn^{2+}-doped QDs with different doping levels (Figure 4f and **Figure S25**). **Figure S25** shows the bleach-recovery dynamics monitored at the band-edge bleach positions. The Mn^{2+} doping concentrations are estimated by their Mn^{2+} PLQY using **Figure S20**. The exciton-to-Mn energy transfer rate is extracted from the bleach recovery dynamics. The average per Mn^{2+} ET rate is highest (~0.4-0.7 ns^{-1}) for lightly doped QDs (~1-2%) and reduces by ~80% (~0.06-0.1 ns^{-1}) for heavily doped (~30-50%) QDs. This estimation aligns with the sublinear correlation between Mn^{2+} PLQY and Mn^{2+} doping concentrations. It is worth noting that our study suggests that the lattice-embedded Mn^{2+} has at least two different chemical environments. The Mn^{2+} ions with different lattice stoichiometry and ligand fields, therefore, may not have the same degree of exchange interaction with excitons.

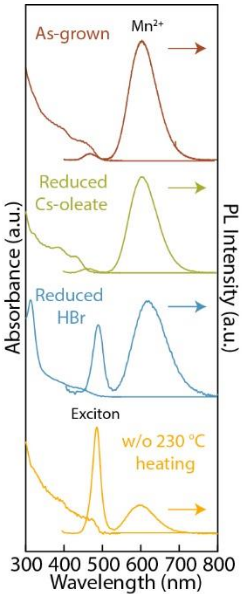

Figure S26. Comparative analysis of Mn^{2+} doping efficiency based on absorption and PL spectra of Mn^{2+}-doped QDs: as-grown (brown), reducing the amount of injected Cs-oleate (green), reducing the amount of HBr added in the reaction mixture (blue), and withdrawing high-temperature heating before Cs-precursor injection (yellow). Reducing the amount of injected Cs-oleate did not significantly affect the Mn^{2+} PLQY. However, using fewer Cs precursors decreased the product's chemical yield. Reducing the amount of HBr will suppress its reaction with Mn-acetate, thereby decreasing the doping efficiency. Additionally, insufficient bromide reduces the colloidal stability of the Mn^{2+}-doped QDs. Removing high-temperature heating significantly reduced the doping efficiency, affecting the further decomposition of Mn^{2+} precursors before Cs-precursor injection.

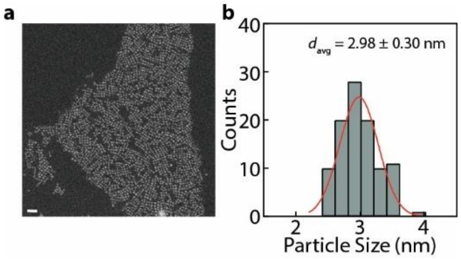

Figure S27. (a) HAADF-STEM image and (b) size distribution histogram with Gaussian fitting curve (red line) for Mn^{2+}-doped $CsPbBr_3$ QDs. The scale bar of the HAADF-STEM image is 20 nm.

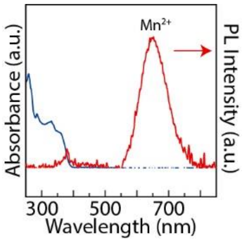

Figure S28. Absorption and PL spectra of highly Mn^{2+}-doped $CsPbCl_3$ QDs. The Mn^{2+} PL peak position is 652 nm, showing a significant redshift (~40 nm) compared with the Mn^{2+} PL peak in Mn^{2+}-doped $CsPbCl_3$ nanocrystals with <1% doping concentration[7]. The redshift is likely a consequence of strong Mn-Mn coupling resulting from a high incorporation of Mn^{2+}.

References

(1) Akkerman, Q. A.; Nguyen, T. P. T.; Boehme, S. C.; Montanarella, F.; Dirin, D. N.; Wechsler, P.; Beiglböck, F.; Rainò, G.; Erni, R.; Katan, C.; et al. Controlling the nucleation and growth kinetics of lead halide perovskite quantum dots. *Science* **2022**, *377* (6613), 1406-1412. DOI: https://doi.org/10.1126/science.abq3616.

(2) Bodnarchuk, M. I.; Boehme, S. C.; ten Brinck, S.; Bernasconi, C.; Shynkarenko, Y.; Krieg, F.; Widmer, R.; Aeschlimann, B.; Günther, D.; Kovalenko, M. V.; et al. Rationalizing and Controlling the Surface Structure and Electronic Passivation of Cesium Lead Halide Nanocrystals. *ACS Energy Lett.* **2019**, *4* (1), 63-74. DOI: https://doi.org/10.1021/acsenergylett.8b01669.

(3) Dong, Y.; Qiao, T.; Kim, D.; Parobek, D.; Rossi, D.; Son, D. H. Precise Control of Quantum Confinement in Cesium Lead Halide Perovskite Quantum Dots via Thermodynamic Equilibrium. *Nano Lett.* **2018**, *18* (6), 3716-3722. DOI: https://doi.org/10.1021/acs.nanolett.8b00861.

(4) Denton, A. R.; Ashcroft, N. W. Vegard's law. *Phys. Rev. A* **1991**, *43* (6), 3161-3164. DOI: https://doi.org/10.1103/PhysRevA.43.3161.

(5) Bailey, R. E.; Nie, S. Alloyed Semiconductor Quantum Dots: Tuning the Optical Properties without Changing the Particle Size. *J. Am. Chem. Soc.* **2003**, *125* (23), 7100-7106. DOI: https://doi.org/10.1021/ja035000o.

(6) Liu, W.; Lin, Q.; Li, H.; Wu, K.; Robel, I.; Pietryga, J. M.; Klimov, V. I. Mn^{2+}-Doped Lead Halide Perovskite Nanocrystals with Dual-Color Emission Controlled by Halide Content. *J. Am. Chem. Soc.* **2016**, *138* (45), 14954-14961. DOI: https://doi.org/10.1021/jacs.6b08085.

(7) Parobek, D.; Roman, B. J.; Dong, Y.; Jin, H.; Lee, E.; Sheldon, M.; Son, D. H. Exciton-to-dopant energy transfer in Mn-doped cesium lead halide perovskite nanocrystals. *Nano Lett.* **2016**, *16* (12), 7376-7380. DOI: https://doi.org/10.1021/acs.nanolett.6b02772.
